# GrowthPredict: A toolbox and tutorial-based primer for fitting and forecasting growth trajectories using phenomenological growth models

**DOI:** 10.1038/s41598-024-51852-8

**Published:** 2024-01-18

**Authors:** Gerardo Chowell, Amanda Bleichrodt, Sushma Dahal, Amna Tariq, Kimberlyn Roosa, James M. Hyman, Ruiyan Luo

**Affiliations:** 1 Department of Population Health Sciences, School of Public Health, Georgia State University, Atlanta, GA USA; 2 School of Medicine, Stanford University, Stanford, CA USA; 3 National Institute for Mathematical and Biological Synthesis (NIMBioS), University of Tennessee, Knoxville, TN USA; 4 Department of Mathematics, Center for Computational Science, Tulane University, New Orleans, LA USA

**Keywords:** Mathematics and computing, Applied mathematics, Software, Statistics, Diseases, Infectious diseases, Computational biology and bioinformatics, Computational models, Programming language, Statistical methods

## Abstract

Simple dynamic modeling tools can help generate real-time short-term forecasts with quantified uncertainty of the trajectory of diverse growth processes unfolding in nature and society, including disease outbreaks. An easy-to-use and flexible toolbox for this purpose is lacking. This tutorial-based primer introduces and illustrates *GrowthPredict*, a user-friendly MATLAB toolbox for fitting and forecasting time-series trajectories using phenomenological dynamic growth models based on ordinary differential equations. This toolbox is accessible to a broad audience, including students training in mathematical biology, applied statistics, and infectious disease modeling, as well as researchers and policymakers who need to conduct short-term forecasts in real-time. The models included in the toolbox capture exponential and sub-exponential growth patterns that typically follow a rising pattern followed by a decline phase, a common feature of contagion processes. Models include the 1-parameter exponential growth model and the 2-parameter generalized-growth model, which have proven useful in characterizing and forecasting the ascending phase of epidemic outbreaks. It also includes the 2-parameter Gompertz model, the 3-parameter generalized logistic-growth model, and the 3-parameter Richards model, which have demonstrated competitive performance in forecasting single peak outbreaks. We provide detailed guidance on forecasting time-series trajectories and available software (https://github.com/gchowell/forecasting_growthmodels), including the full uncertainty distribution derived through parametric bootstrapping, which is needed to construct prediction intervals and evaluate their accuracy. Functions are available to assess forecasting performance across different models, estimation methods, error structures in the data, and forecasting horizons. The toolbox also includes functions to quantify forecasting performance using metrics that evaluate point and distributional forecasts, including the weighted interval score. This tutorial and toolbox can be broadly applied to characterizing and forecasting time-series data using simple phenomenological growth models. As a contagion process takes off, the tools presented in this tutorial can help create forecasts to guide policy regarding implementing control strategies and assess the impact of interventions. The toolbox functionality is demonstrated through various examples, including a tutorial video, and the examples use publicly available data on the monkeypox (mpox) epidemic in the USA.

## Introduction

Reliable short-term forecasts of time series describing an evolving transmission or growth process are essential for decision-making in all aspects of life, including predicting the weather, commercial product demand, the number of cases of an emerging infectious disease, and the growth or decline of the economy. Simple statistical models such as autoregressive integrated moving average (ARIMA) models have been popular for forecasting time series^1–5^. In contrast, dynamical models based on rates of change equations (i.e., differential equations) are less frequently applied by non-specialists in specific scientific fields. However, forecasts based on dynamic models can provide more information about the process of interest by characterizing specific mechanisms and parameters involved in their dynamics^6–9^. For instance, the simple phenomenological growth models discussed in this tutorial can help characterize growth rates, scaling of growth, doubling times, reproduction numbers, and turning points, as well as predicting the size of a growing population (i.e., carrying capacity) or an epidemic outbreak (i.e., epidemic size) at different time horizons with quantified uncertainty^10–17^. Hence, there is a need for an easy-to-use and flexible toolbox to generate short-term forecasts from simple phenomenological growth models with quantified uncertainty of the trajectory of diverse growth processes observed in nature and society, such as infectious disease outbreaks^6^.

This tutorial paper introduces a user-friendly MATLAB toolbox to fit and forecast time-series trajectories of infectious diseases using phenomenological dynamic growth models based on ordinary differential equations (ODEs) that will find broad applications in the natural and social sciences. This toolbox is written for various audiences, including students training in time-series forecasting, dynamic growth modeling, parameter estimation, parameter uncertainty and identifiability, model comparison, performance metrics, and forecast evaluation. The toolbox is also helpful for researchers and policymakers who need to conduct short-term forecasts by relying on historical and real-time trajectory data of the process of interest, such as an unfolding epidemic.

This forecasting toolbox utilizes a variety of phenomenological growth models based on ordinary differential equations (ODEs), such as the generalized-logistic growth model (GLM) and the Richards model, which have shown competitive performance in modeling epidemic outbreaks in prior studies^10,18,19^. These models capture exponential and sub-exponential growth patterns that typically follow a rising pattern followed by a decline phase, a common feature of contagion processes^10,14,15,20^.

Specifically, models in the toolbox include the 2-parameter generalized-growth model (GGM)^21,22^, which has proved useful in characterizing and forecasting the ascending phase of epidemic outbreaks^18^, the 2-parameter Gompertz model, the 3-parameter GLM, and the Richards model, which have each demonstrated competitive performance in forecasting single peak epidemics^10,18,19^. The toolbox includes nonlinear least squares estimation (LSQ) and maximum likelihood estimation (MLE) methods with different assumptions about the error structure of the observed data, including normal, Poisson, and negative binomial distributions, as well as uncertainty quantification based on a parametric bootstrapping approach^6,19^. The toolbox also includes functions to quantify forecasting performance using metrics that evaluate point and distributional forecasts, including the weighted interval score (WIS). The toolbox’s functions are illustrated using weekly publicly available case data from the monkeypox (mpox) epidemic in the USA.

This tutorial-based primer is organized as follows. After providing an overview of the toolbox functions for users, we introduce the parameter estimation methods included in the toolbox and then describe the underlying methodology and user parameters and functions to calibrate, evaluate, and display model fits to data. Finally, we use specific examples in the context of the monkeypox epidemic in the USA to demonstrate the functions that generate, display, and quantify the performance of model-based forecasts, as well as estimate doubling times and the effective reproduction number. A tutorial video that demonstrates the toolbox functionality is available at: https://www.youtube.com/channel/UC6IzIu-pPcMLlLYAho43loQ.

## Implementation

### Installing the toolbox

- Download the MATLAB code located in folder ‘**forecasting_growthmodels code**’ from the GitHub repository: https://github.com/gchowell/forecasting_growthmodels
- Create ‘input’ folder in your working directory where your input data will be stored.
- Create ‘output’ folder in your working directory where the output files will be stored.
- Open a MATLAB session.

### Overview of the toolbox functions

Table 1 lists the names of user functions associated with the toolbox, along with a brief description of their role. The internal functions associated with the toolbox are given in supplementary file 1 (Table 1S). The user needs to specify the parameters related to model fitting and forecasting in the options_fit.m and options_forecast.m files.

**Table 1:** Description of the user functions associated with the toolbox.

Function	Role
options_fit.m	Specifies the parameters related to model fitting, including the characteristics of the time series data, the model, the parameter estimation method, the error structure, and the calibration period. The structure of the options_fit.m file is described in Text 1S (supplementary file 1)
options_forecast.m	Specifies the parameters related to model forecasting, including the characteristics of the time series data, the model, the parameter estimation method, the error structure, the calibration period, and forecasting period. The structure of the options_forecast.m file is described in Text 2S (supplementary file 1)
options_Rt.m	Specifies the parameters related to the estimation of the effective reproduction number, namely the type of generation interval distribution and the mean and variance of the generation interval for the infectious disease of interest
plotGrowthModel.m	Plots model solutions where the user provides the type of growth model, parameter values, and the initial condition
Run_Fit_GrowthModels.m	Fits a model to data with quantified uncertainty
Run_Forecasting_GrowthModels.m	Fits a model to data with quantified uncertainty and generates a model-based forecast with quantified uncertainty
plotFit_GrowthModels	Displays the model fit and the empirical distribution of the estimated parameters. It also saves output .csv files in the output folder with the model fit, the parameter estimates with 95% confidence intervals (CIs), the calibration performance metrics, and the doubling times estimated from the incidence trajectory
plotForecast_GrowthModels	Displays the model-based forecast and the associated performance metrics. Moreover, the data associated with the forecasts, the parameter estimates, the doubling times estimated from the incidence trajectory, and the calibration and forecasting performance metrics, are saved as .csv files in the output folder
plotFit_ReproductionNumber	Displays the model fit and the effective reproduction number. It also saves .csv files in the output folder with the model fit, the parameter estimates with 95% CIs, the calibration performance metrics, and the doubling times estimated from the incidence trajectory
plotForecast_ReproductionNumber	Displays the model-based forecast and the corresponding effective reproduction number. Moreover, the data associated with the forecasts, the parameter estimates, the doubling times estimated from the incidence trajectory, and the calibration and forecasting performance metrics, are saved as .csv files in the output folder

### Overview of the tutorial

**Figure 1:**
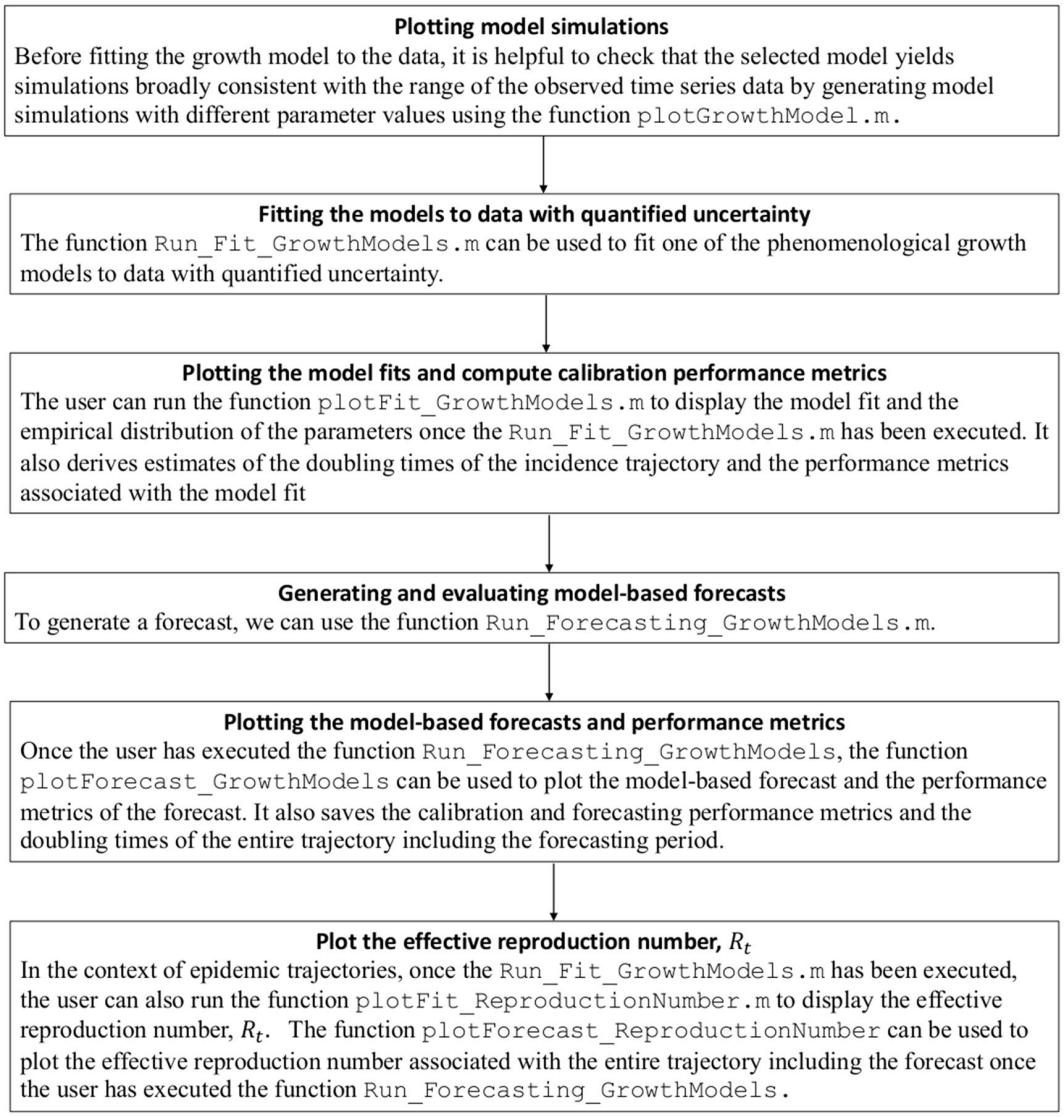
Overview of the workflow for fitting and forecasting time series trajectories described in this tutorial.

The workflow described in this tutorial, summarized in Fig. 1, is composed of 6 main sections: (1) plotting preliminary model simulations, (2) fitting the models to data through statistical inference, (3) plotting the resulting model fits, (4) generating short-term forecasts with quantified uncertainty, (5) plotting the resulting short-term forecasts and the associated performance metrics, and (6) calculating the effective reproduction number in the context of epidemic time series.

### Parameter estimation method

Let ${y}_{{t}_{1}, }{y}_{{t}_{2}},\dots ,{y}_{{t}_{{n}_{d}}}$ denote the time series of the epidemic trajectory used in calibrating the model. Here, ${t}_{j}$, $j=\mathrm{1,2},\dots ,{n}_{d}$, are the time points for the time series data, and ${n}_{d}$ is the number of observations. Let $f\left(t,\Theta \right)$ denote the expected curve of the epidemic trajectory. We can estimate the set of model parameters, denoted by $\Theta$, by fitting the model solution to the observed data via nonlinear least squares (LSQ)^23^; within the MATLAB toolbox, this is realized by setting the parameter <method1> to 0 in the options_fit.m or options_forecast.m files. Least squares estimation is achieved by searching for the set of parameters, $\Theta$, that minimizes the sum of squared differences between the observed data ${y}_{{t}_{1}, }{y}_{{t}_{2}}\dots ..{y}_{{t}_{{n}_{d}}}$ and the best fit of the model (model mean) which corresponds to $f(t,\Theta )$. That is, $\Theta$ is estimated by $\widehat{\Theta }={\text{argmin}} \sum_{j=1}^{{n}_{d}}{(f\left({t}_{j},\Theta \right)-{y}_{{t}_{j}})}^{2}$. In the following section, we describe different phenomenological growth models included in this toolbox for the expected epidemic trajectory curve $f(t,\Theta )$.

This parameter estimation method (LSQ) weights each data point equally and does not require a specific distributional assumption for ${y}_{t}$, except for the first moment $E[{y}_{t}]=f(t;\Theta )$. That is, the mean of the observed data at time *t* equals the expected count denoted by $f\left(t,\Theta \right)$ at time *t*^24^. Under mild regularity conditions, this method yields asymptotically unbiased point estimates regardless of any misspecification of the variance–covariance error structure. Hence, the estimated model $f(t,\widehat{\Theta })$ best fits the mean of the observed data in terms of the L2 norm. We can use the *fmincon* function in MATLAB to set the optimization problem. Finally, we also employ MATLAB’s **MultiStart** feature to specify the number of random initial guesses of the model parameters using the parameter <numstartpoints> in the options_fit.m or options_forecast.m files to thoroughly search for the best-fit parameter estimates.

In addition to nonlinear least squares fitting, we can also estimate model parameters via maximum likelihood estimation (MLE)^25^ with specific assumptions about the error structure in the data (e.g., Poisson, Negative binomial) via parameter <method1> . The log-likelihood expressions derived for Poisson and negative binomial error structures are given below.

- Poisson

For a Poisson error structure, the full log-likelihood of Poisson (<method1>=1) is given by:

$$\sum_{i=1}^{n}\left\{{y}_{i}ln({\mu }_{i})-ln({y}_{i}!)-{\mu }_{i}\right\}$$

where ${\mu }_{i}=f({t}_{i},\Theta )$ denotes the mean of ${y}_{i}$ at time ${t}_{i}$ and $f({t}_{i},\Theta )$ is the mean curve to be estimated from the differential equation.

(b) Negative binomial

Let *r* $>$ 0 denote the number of failures until the experiment is stopped and $p\in [\mathrm{0,1}]$ denote the success probability in each experiment. Then the number of successes, *y,* before the *r*-th failure occurs has a **negative binomial** distribution:

$$f\left(y|r,p\right)=\left(\genfrac{}{}{0pt}{}{r+y-1}{y}\right){p}^{y }{(1-p)}^{r}=\frac{1}{y!}\prod_{j=0}^{y-1}\left(j+r\right) .{p}^{y}({1-p)}^{r}$$

with ${\text{mean}}=\mu =\frac{rp}{(1-p)}$ and variance=${\sigma }^{2}=\frac{rp}{{\left(1-p\right)}^{2}}> \mu .$ For n observations ${y}_{1}$, …,${y}_{n}$, the full log-likelihood is

1.1 $$l\left(r,p\right)=\sum_{i=1}^{n}\left\{\left\{\sum_{j=0}^{{y}_{i}-1}ln(j+r)\right\} +{y}_{i}ln(p)+rln(1-p)-ln({y}_{i}!)\right\}.$$

We can express the likelihood with $\mu$ and *σ*^2^ by substituting $p=1-\frac{\upmu }{{\upsigma }^{2}}$ and $r=\frac{{\mu }^{2}}{{\sigma }^{2}-\mu }$, where $\mu =f (t,\Theta )$ is the mean curve to be estimated from the differential equation.

There are different types of variances commonly used in a negative binomial distribution. If the variance scales linearly with the mean, then ${\sigma }^{2}=\mu +\alpha \mu ,$(i.e., <method1>=3 in options_fit.m or options_forecast.m), $p=\frac{\alpha }{1+\alpha }$ and $r=\mu /\alpha$. The full log-likelihood (1.1) can be expressed as follows:

1.2 $$l\left(\Theta ,\alpha \right)=\sum_{i=1}^{n}\left\{\left\{\sum_{j=0}^{{y}_{i}-1}ln(j+{\alpha }^{-1}f({t}_{i},\Theta ))\right\} +{y}_{i}ln(\alpha )-({y}_{i}+{\alpha }^{-1}f({t}_{i},\Theta ))ln(1+\alpha )-ln({y}_{i}!)\right\}.$$

If the variance scales quadratically with the mean, then ${\sigma }^{2}=\mu +\alpha {\mu }^{2}$ (ie., <method1>=4 in options_fit.m or options_forecast.m), $p=\frac{\alpha \mu }{1+\alpha \mu }$ and $r=1/\alpha .$ The full log-likelihood (1.1) can be expressed as follows:

1.3 $$l\left(\Theta ,\alpha \right)=\sum_{i=1}^{n}\left\{\left\{\sum_{j=0}^{{y}_{i}-1}ln(j+{\alpha }^{-1})\right\} +{y}_{i}ln(\alpha f({t}_{i},\Theta ))-({y}_{i}+{\alpha }^{-1})ln(1+\alpha f({t}_{i},\Theta ))-ln({y}_{i}!)\right\}.$$

The more general form of variance is ${\sigma }^{2}=\mu +\alpha {\mu }^{d}$ (i.e., <method1>=5 in options_fit.m or options_forecast.m) with any $-\infty <d<\infty$. Then the full log-likelihood (1.1) can be expressed as follows:

1.4 $$l\left(\Theta ,\alpha \right)=\sum_{i=1}^{n}\left[\left\{\sum_{j=0}^{{y}_{i}-1}ln(j+{\alpha }^{-1}{{\mu }_{i}}^{2-d})\right\}+{y}_{i}ln(\alpha {{\mu }_{i}}^{d-1})-({y}_{i}+{\alpha }^{-1}{{\mu }_{i}}^{2-d})ln(1+\alpha {{\mu }_{i}}^{d-1})-ln({y}_{i}!)\right]$$

where $${\mu }_{i}=f ({t}_{i},\Theta ).$$

Finally, it is worth noting that the above full log-likelihood expressions allow the selection or comparison of models based on different error structures via their AIC_c_ (corrected Akaike Information Criterion) values. However, if the goal is to focus on different models with the same type of error structure (e.g., normal), we could use simplified likelihood expressions by removing the constants to speed up running time.

### Parametric bootstrapping

To quantify parameter uncertainty, we follow a parametric bootstrapping approach, which allows the computation of standard errors and related statistics without closed-form formulas^26^. We generate $B$ bootstrap samples from the best-fit model $f(t,\widehat{\Theta })$, with an assumed error structure specified using parameter <dist1> in the options_fit.m or options_forecast.m files, to quantify the uncertainty of the parameter estimates and construct confidence intervals. The bootstrapping algorithm is given in ref^6^. Typically, the error structure in the data is modeled using a probability model such as the normal, Poisson or negative binomial distribution. Using nonlinear least squares (<method1>=0), in addition to a normally distributed error structure (<dist1>=0), we can also assume a Poisson (<dist1>=1) or a negative binomial distribution (<dist1>=2), whereby the variance-to-mean ratio is empirically estimated from the time series. To estimate this constant ratio, we group a fixed number of observations (e.g., 7 observations for daily data into a bin across time), calculate the mean and variance for each bin, and then estimate a constant variance-to-mean ratio by calculating the average of the variance-to-mean ratios over these bins. For non-normal error structure, we estimate parameters using maximum likelihood estimation (MLE) assuming Poisson or negative binomial error structures in the data (<method1>=1 & <dist1>=1 for Poisson and  <method1>=3,  <method1>=4 & <dist1>=4, and <method1>=5 & <dist1>=5 for the different negative binomial error structures described above).

Specifically, using the best-fit model $f(t,\widehat{\Theta })$, we generate *B*-times replicated simulated datasets of size ${n}_{d}$, where the observation at time ${t}_{j}$ is sampled from the corresponding distribution specified by <dist1>. Next, we refit the model to each of the *B* simulated bootstrap datasets and re-estimate the parameters using the same estimation method as for the original data. The new parameter estimates for each realization are denoted by ${\widehat{\Theta }}_{b},$ where $b=\mathrm{1,2},\dots ,B.$ Using the *B* sets of re-estimated parameters $\left({\widehat{\Theta }}_{b}\right),$ we can characterize the empirical distribution of each parameter estimate, calculate the variance, and construct confidence intervals for each parameter. The resulting uncertainty around the model fit can similarly be obtained from $f\left(t,{\widehat{\Theta }}_{1}\right),$ $f\left(t,{\widehat{\Theta }}_{2}\right),\dots ,f(t,{\widehat{\Theta }}_{B})$. For the purposes of this tutorial, we characterize the uncertainty using 300 bootstrap realizations (i.e., parameter < B >  = 300 in the options_fit.m or options_forecast.m files).

### Model-based forecasts with quantified uncertainty

Based on the best-fit model $f\left(t,\widehat{\Theta }\right),$ we can forecast $h$ days ahead based on the estimate $f(t+h,\widehat{\Theta })$. The uncertainty of the forecasted value can be obtained using the previously described parametric bootstrap method. Let

$$f\left(t+h,{\widehat{\Theta }}_{1}\right), f\left(t+h,{\widehat{\Theta }}_{2}\right),\dots ,f(t+h,{\widehat{\Theta }}_{B})$$

denote the forecasted value of the current state of the system propagated by a horizon of *h* time units, where ${\widehat{\Theta }}_{b}$ denotes the estimation of parameter set $\Theta$ from the *b*_*th*_ bootstrap sample. We can use these values to calculate the bootstrap variance to measure the uncertainty of the forecasts and use the 2.5% and 97.5% percentiles to construct the 95% prediction intervals (95% PI). We can set the forecasting horizon (*h*) using the parameter  <forecastingperiod1> in the options_forecast.m file.

For illustration, we fit the models through LSQ with a normal error structure (i.e., <method1>=0 and  <dist1>=0) for the monkeypox data. In the options_fit.m or options_forecast.m files, the values of the parameters related to the parameter estimation method and parametric bootstrapping follow:
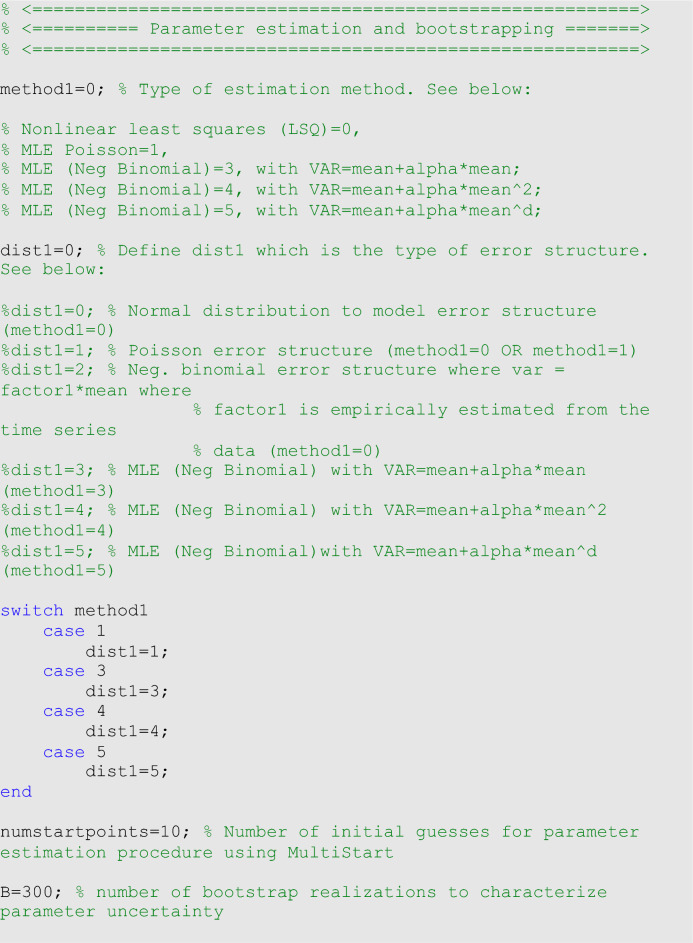


### Phenomenological growth models

Below we describe the growth models included in the toolbox. We use $C\left(t\right)$ to denote the cumulative case count at time $t$ and ${C}^{\prime}\left(t\right)$ denotes the expected epidemic trajectory curve $f\left(t,\Theta \right).$

- Generalized-growth model (GGM)

Models commonly used to study the growth pattern of infectious disease outbreaks often assume exponential growth in the absence of control interventions (e.g., compartmental models); however, growth patterns are likely slower than exponential for some diseases, depending on the transmission mode and population structure. For example, Ebola spreads via close contact, therefore, sub-exponential growth patterns would be expected in a constrained population contact structure^27^. The generalized growth model (GGM)^21^ includes a “deceleration of growth” parameter, *p* (range: [0, 1]), that relaxes the assumption of exponential growth. A value of $p=0$ represents constant (linear) growth, while a value of $p=1$ indicates exponential growth. If $0<p<1$, the growth pattern is characterized as sub-exponential or polynomial.

The GGM is as follows:

$$\frac{dC(t)}{dt}={C}^{\prime}\left(t\right)=rC{(t)}^{p},$$

where the derivative $C^{\prime}(t)$ describes the incidence curve over time *t*. The positive parameter $r$ is the growth rate parameter ($r>0$), and $p$ is the scaling of growth parameter^21^. For this model, we estimate $\Theta =\left(r,p\right).$ This model can be selected by setting <flag1>=0 in the options_fit.m and options_forecast.m files.

(b) Generalized logistic growth model (GLM)

The generalized logistic growth model (GLM) has three parameters and is given by:

$$\frac{dC(t)}{dt}={C}^{\prime}\left(t\right)=r{C}^{p}(t)\left(1-\frac{C(t)}{{K}_{0}}\right)$$

The growth scaling parameter, $p$, is also used in the GLM to model a range of early epidemic growth profiles ranging from constant incidence $\left(p=0\right),$ polynomial $0<{\text{p}}<1,$ and exponential growth dynamics $(p=1)$. When $p=1$, this model reduces to the logistic growth model (< flag1 >  = 3). The remaining model parameters are as follows: ${\text{r}}$ is the growth rate, and ${K}_{0}$ is the final cumulative epidemic size. For this model, $\Theta =(r,p,{K}_{0})$. The GLM has been employed to generate short-term forecasts of Zika, Ebola, and COVID-19 epidemics^10,14,15,28^. This model can be selected by setting <flag1>=1 in the options_fit.m and options_forecast.m files.

(c) Richards model (RIC)

The well-known Richards model extends the simple logistic growth model and relies on three parameters. It extends the simple logistic growth model by incorporating a scaling parameter, $a$, that measures the deviation from the symmetric simple logistic growth curve^6,29,30^. The Richards model is given by the differential equation:

$$\frac{dC\left(t\right)}{dt}=rC(t)\left[1-{\left(\frac{C(t)}{{K}_{0}}\right)}^{a}\right]$$

where  $r$ is the growth rate, $a$ is a scaling parameter and ${K}_{0}$ is the final epidemic size. The Richards model has been employed to generate short-term forecasts of SARS, Zika, Ebola, and COVID-19 epidemics^10,11,14,15,28^. For this model, we estimate $\Theta =(r,a,{K}_{0})$. This model can be selected by setting <flag1>=4 in the options_fit.m and options_forecast.m files. A 4-parameter extension of the Richards model is the generalized Richards model (<flag1>=2), which incorporates the growth scaling parameter $p$ used in the GGM and GLM.

(d) Gompertz model (GOM)

The 2-parameter Gompertz model is given by:

$$\frac{dC(t)}{dt}={C}^{\prime}\left(t\right)=rC(t){e}^{-bt}$$

where $r$ is the growth rate and $b>0$ describes the exponential decline of the growth rate. For this model, we estimate $\Theta =\left(r,b\right).$ The GOM model has been employed to generate short-term forecasts of Zika and COVID-19 epidemics^17,31,32^. This model can be selected by setting <flag1>=5 in the options_fit.m and options_forecast.m files.

### Initial condition

Besides the parameters of the dynamic growth models, it is also possible to estimate the initial number of cases in the time series, rather than fixing $C(0)$ according to the first data point in the time series by specifying the Boolean variable <fxI0> in the options_fit.m and options_forecast.m files. Specifically, <fixI0>=1 fixes the initial condition according to the first data point in the time series, whereas <fixI0>=0 estimates the initial condition along the other model parameters.

### Quality of model fit

To assess the quality of model fit, we can compare the *AIC*_*c*_ (corrected Akaike Information Criterion) values of the best-fit models. The ${AIC}_{c}$ is given by^33,34^:

$${AIC}_{c}=-2log\left(likelihood\right)+2m+\frac{2m\left(m+1\right)}{{n}_{d}-m-1}$$

where $m$ is the number of model parameters and ${n}_{d}$ is the number of data points. Specifically for normal distribution, the ${AIC}_{c}$ is

$${AIC}_{c}={n}_{d}log\left(SSE\right)+2m+\frac{2m\left(m+1\right)}{{n}_{d}-m-1}$$

where $SSE=\sum_{j=1}^{{n}_{d}}{(f\left({t}_{j},\widehat{\Theta }\right)-{y}_{{t}_{j}})}^{2}$. Thus, this metric accounts for model complexity regarding the number of model parameters and is used for model selection. In the options_fit.m and options_forecast.m files, the values of the parameters related to the selection of the growth model follow:


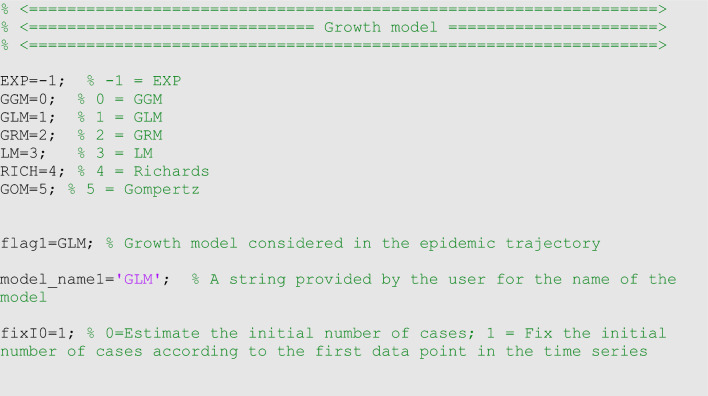


### Plotting simulations of the growth model

Before fitting the growth model to the data, it is helpful to check that the selected model yields simulations broadly consistent with the range of the time series data by generating model simulations with different parameter values. For example, if data show systematic differences that contrast with the model solutions (e.g., multiple peaks or plateaus), it may suggest that the model is not the best choice for the data at hand.

The function plotGrowthModel.m can be used to plot model solutions where the user provides the type of growth model, parameter values, and initial condition as passing input parameters to the function in the following order: <flag1>, $r$, $p$, $a,K,C\left(0\right),$ and finally the duration of the simulation (i.e., the time span over which the model is numerically solved). For example, the following call plots a simulation of the GLM model (<flag1>=1) with the following parameter values: $r=0.18, p=0.9,$ and $K=1000.$ The initial condition is indicated by $C\left(0\right)=1$, and the total duration of the simulation is set at $200$.

 > > **plotGrowthModel(**1,0.18,0.9,[],10000,1,200**)**

**Figure 2:**
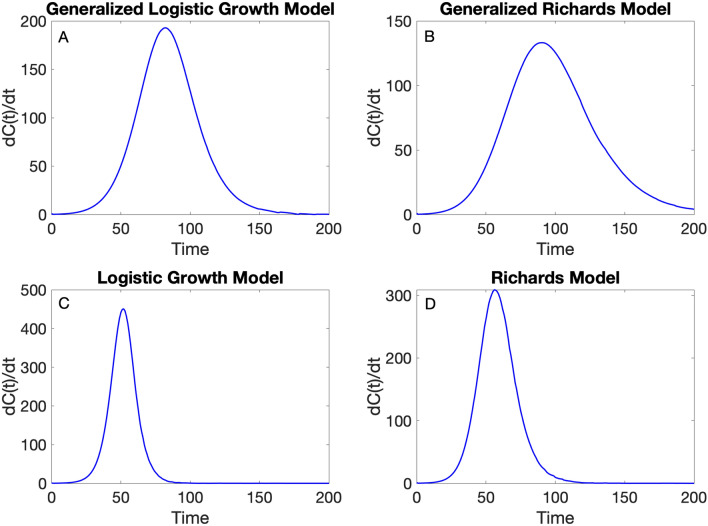
Representative simulations from various growth models, namely (**A**) <flag1>=1, (**B**) <flag1>=2, (**C**)  <flag1>=3, and (**D**)  <flag1>=4, supported in the toolbox using parameter values: $r=0.18,p=0.9,a=0.6, K=10000.$ Here, $r$ refers to the growth parameter, $p$ is a growth scaling parameter (GLM and GGM models), and $a$ is a scaling parameter employed with the Richards model. $K$ refers to the size of the epidemic. The initial condition is $C\left(0\right)=1$, and the total duration of the simulation is set at $200$.

Of note, in the above call, the value of parameter $a$ is passed empty ([]) since the GLM model does not use this parameter. This function will generate a figure (Fig. 2A) that shows the corresponding model solution, $dC(t)/dt$. Additional representative simulations from other growth models supported in the toolbox are shown in Fig. 2

### Model and code testing

**Figure 3:**
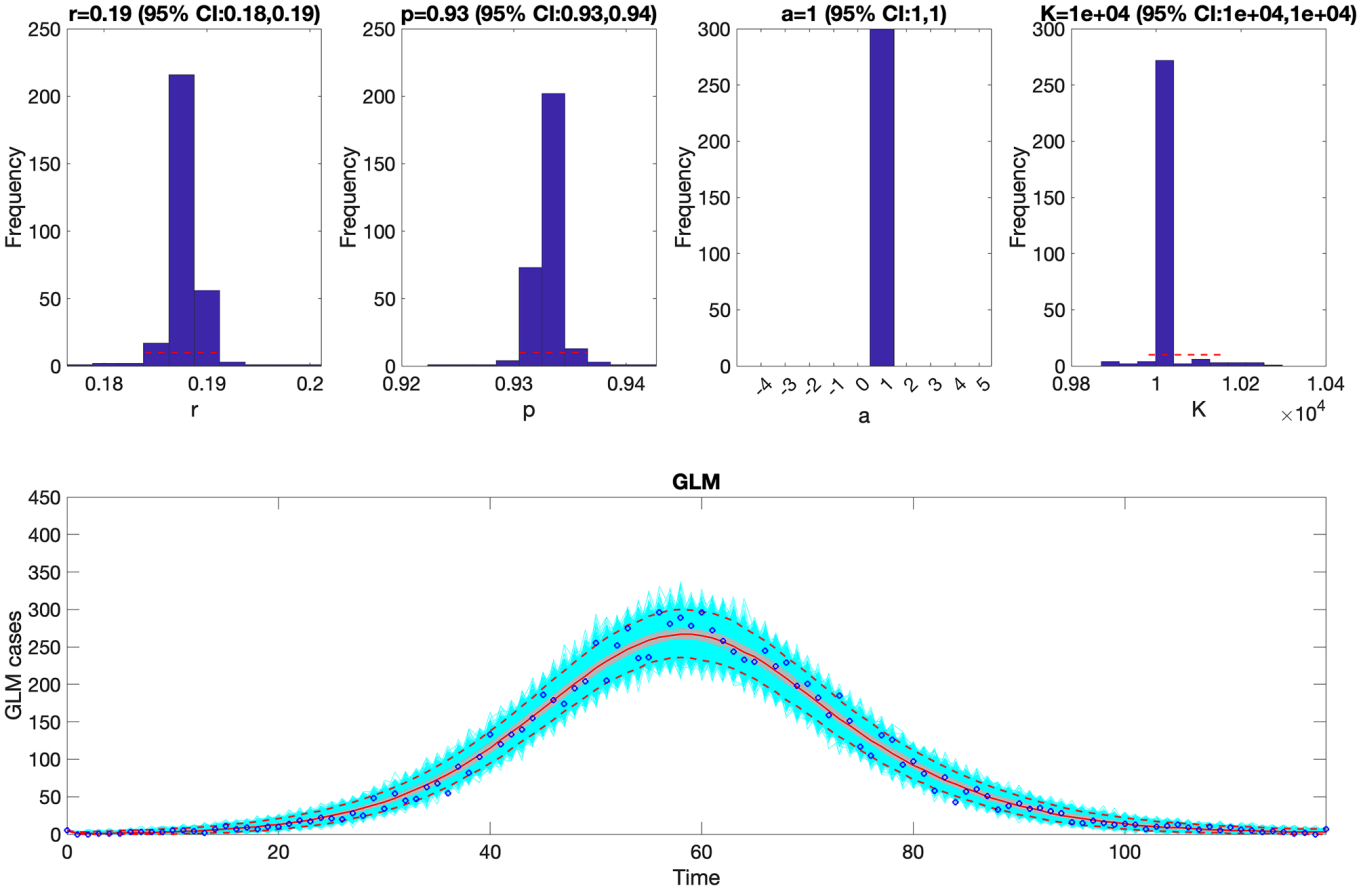
The generalized logistic-growth model (GLM) fit, and the empirical distribution of the estimated parameters for the simulated data. These findings indicate that the parameter estimates of $r$ (growth rate), $p$ (growth scaler) and $K$ (epidemic size) are in line with the “true” parameter values used to simulate the data. The scaling parameter, $a$, is not estimated when employing the GLM. In the bottom panel, the solid red line is the median model fit and the dashed lines correspond to the 95% prediction intervals. The blue dots indicate the observed data points. The gray lines correspond to the mean of the model fits obtained from the parametric bootstrapping with 300 bootstrap realizations, and the cyan lines indicate the predictive uncertainty around the model fit.

Before fitting the model to the data, it is helpful to check that the code works properly with simulated data before applying the toolbox to actual data. If we can estimate the true parameter values used to generate simulated data from the model, we can confidently move forward that the code is working appropriately with simulated data. For this purpose, we used the following selected values $r=0.16, p=0.94$ and $K=10000,$ to generate a dataset from the GLM with Poisson error structure, initial condition $C\left(0\right)=1$, and duration of 120 days. We then proceeded to estimate the three model parameters for the simulated data using maximum likelihood (i.e., <method1>=1, <dist1>=1). The corresponding options_fit.m file is given in supplementary file 1 (Code File 1S). Figure 3 shows the GLM fit and the empirical distribution of the estimated parameters for the simulated data. These findings indicate that the parameter estimates align with the “true” parameter values used to simulate the data. Moreover, the model is well calibrated to the data with the 95% prediction interval coverage falling at 95%.

### Performance metrics

To assess calibration and forecasting performance, we used four performance metrics: the mean absolute error (MAE), the mean squared error (MSE), the coverage of the 95% prediction intervals (95% PI), and the weighted interval score (WIS)^35^. While it is possible to generate *h*-time units ahead forecasts of an evolving process, those forecasts looking into the future can only be evaluated when sufficient data for the *h*-time units ahead has been collected. In the options_forecast.m file, the parameter <getperformance> is a Boolean variable (0/1) to indicate whether the user wishes to compute the performance metrics of the forecasts when sufficient data is available.

The *mean absolute error* (MAE) is given by:

$$\text{MAE= }\frac{1}{N}\sum_{h=1}^{N}\left|f\left({t}_{h},\widehat{\Theta }\right)-{y}_{{t}_{h}}\right|,$$

where ${t}_{h}$ are the time points of the time series data^36^, and *N* is the calibration or forecasting period length. Similarly, the *mean squared error* (MSE) is given by:

$$\text{MSE = }\frac{1}{N}\sum_{h=1}^{N}{(f({t}_{h},\widehat{\Theta })-{y}_{{t}_{h}})}^{2} .$$

The coverage of the *95% prediction interval (95% PI*) corresponds to the fraction of data points that fall within the 95% PI, and is calculated as

$${95}\% \,{\text{PI}}\,{\text{coverage}} = \frac{1}{N}\mathop \sum \limits_{t = 1}^{N} {\mathbf{1}}\{ Y_{t} > L_{t} \cap Y_{t} < U_{t} \},$$

where ${L}_{t}$ and ${U}_{t}$ are the lower and upper bounds of the 95% PIs, respectively, ${Y}_{t}$ are the data and **1** is an indicator variable that equals 1 if ${Y}_{t}$ is in the specified interval and 0 otherwise.

The *weighted interval score* (WIS)^35,37^, which is a proper score recently embraced for quantifying model forecasting performance in epidemic forecasting studies^38–42^, provides quantiles of predictive forecast distribution by combining a set of Interval Score (IS) for probabilistic forecasts. An IS is a simple proper score that requires only a central $\left(1-\alpha \right)*100\%$ PI^35^ and is described as

$${IS}_{\alpha }\left(F,y\right)=\left(u-l\right)+\frac{2}{\alpha }\times \left(l-y\right)\times 1\left(y<l\right)+\frac{2}{\alpha }\times \left(y-u\right)\times 1\left(y>u\right) .$$

In this Eq. 1 refers to the indicator function, meaning that $1\left(y<l\right)=1$ if $y<l$ and 0 otherwise. The terms $l$ and $u$ represent the $\frac{\alpha }{2}$ and $1-\frac{\alpha }{2}$ quantiles of the forecast $F.$ The IS consists of three distinct quantities:

1. The sharpness of $F,$ given by the width $u-l$ of the central $\left(1-\alpha \right) \times 100\%$ PI.
2. A penalty term $\frac{2}{\alpha }\times \left(l-y\right)\times 1\left(y<l\right)$ for the observations that fall below the lower end point $l$ of the $\left(1-\alpha \right)\times 100\%$ PI. This penalty term is directly proportional to the distance between $y$ and the lower end $l$ of the PI. The strength of the penalty depends on the level $\alpha .$
3. An analogous penalty term $\frac{2}{\alpha }\times \left(y-u\right)\times 1\left(y>u\right)$ for the observations falling above the upper limit $u$ of the PI.

To provide more detailed and accurate information on the entire predictive distribution, we report several central PIs at different levels $\left( {1 - \alpha _{1} } \right) < \left( {1 - \alpha _{2} } \right) < \cdots < \left( {1 - \alpha _{K} } \right)$ along with the predictive median, $\widetilde{y}$, which can be seen as a central prediction interval at level $1-{\alpha }_{0} \to 0$. This is referred to as the WIS, and it can be evaluated as follows:

$$WI{S}_{{\alpha }_{0:K}}\left(F,y\right)=\frac{1}{K+\frac{1}{2}}.({w}_{0}.\left|y-\widetilde{y}\right|+{\sum }_{k=1}^{K}{w}_{k}.I{S}_{{\alpha }_{k}}\left(F,y\right))$$

where, ${w}_{k}=\frac{{\alpha }_{k}}{2}$ for $k=\mathrm{1,2},\dots .K$ and ${w}_{0}=\frac{1}{2}$. Hence, WIS can be interpreted as a measure of how close the entire distribution is to the observation in units on the scale of the observed data^39,43^.

In the options_forecast.m file, we can specify the parameters related to the epidemic forecasts, including the forecasting horizon and an indicator variable to specify whether the forecasting performance metrics should be computed:
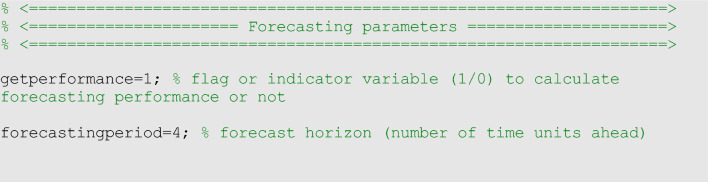


### Doubling times

Doubling times characterize the sequence of times at which the cumulative incidence doubles. Denote the times at which cumulative incidence doubles by ${t}_{{d}_{j}}$, such that ${2C(t}_{{d}_{j}})={C(t}_{{d}_{j+1}})$ where ${t}_{{d}_{0}}=0, C\left( {t}_{{d}_{0}}\right)={C}_{0}$, $j=\mathrm{1,2},3,\dots ,{n}_{g}$ and ${n}_{g}$ is the total number of times cumulative incidence doubles^44,45^. The actual sequence of “doubling times” is defined as follows:

$${d}_{j}={\Delta t}_{{d}_{j}}={t}_{{d}_{j}}-{t}_{{d}_{j-1}}\mathrm{where }j=\mathrm{1,2},3,\dots ,{n}_{g}.$$

For exponential growth, doubling times remain invariant and are given by $({\text{ln}}2)/r$, whereas the doubling times increase when the growth pattern follows sub-exponential growth^44^. We can characterize the doubling times and their uncertainty from the best-fit model $f\left(t,\widehat{\Theta }\right)$^46^. We can evaluate the uncertainty of the sequence of doubling times and the overall doubling time using the model parameter estimates derived from bootstrapping $\left({\widehat{\Theta }}_{b}\right),$ where $b=\mathrm{1,2},3,\dots ,B$. That is, ${d}_{j}({\widehat{\Theta }}_{b})$ provides a sequence of doubling times for a set of bootstrap parameter estimates, ${\widehat{\Theta }}_{b}$, where $b=\mathrm{1,2},3,\dots ,B$. We can use these curves to derive 95% CIs for the sequence of doubling times and quantify the probability of observing a given number of doublings.

### The effective reproduction number, ${{\text{R}}}_{{\text{t}}}$

While the basic reproduction number, commonly denoted by ${R}_{0}$, gauges the transmission potential at the onset of an epidemic^47^, the effective reproduction number, ${R}_{t}$, captures changes in transmission potential throughout the epidemic^48,49^. We can characterize the effective reproduction number and its uncertainty from the best-fit model $f\left(t,\widehat{\Theta }\right)$^46^. We can derive the 95% CI of ${R}_{t}$ from the uncertainty associated with the parameter estimates derived from bootstrapping $({\widehat{\Theta }}_{b})$ where $b=\mathrm{1,2},3,\dots ,B$. That is, ${R}_{t}({\widehat{\Theta }}_{b})$ provides a curve of the effective reproduction number for a set of parameter values ${\widehat{\Theta }}_{b}$ where $b=\mathrm{1,2},3,\dots ,B$. Denote the incidence at calendar time ${t}_{j}$ by $I({t}_{j}, {\widehat{\Theta }}_{b})$, and the discretized probability distribution of the generation interval by ${\rho }_{{t}_{j}}$. The effective reproduction number ${R}_{t}({\widehat{\Theta }}_{b})$ can be estimated using the renewal equation^48,49^:

$${R}_{{t}_{j}}\left({\widehat{\Theta }}_{b}\right)=\frac{I\left({{t}_{j,}\widehat{\Theta }}_{b}\right)}{\sum_{k=0}^{j}I\left({{t}_{j-k,}\widehat{\Theta }}_{b}\right){\rho }_{{t}_{k}}}$$

where the denominator represents the total number of cases that contribute (as primary cases) to generate the number of new cases ${I}_{{t}_{j}}$ (as secondary cases) at calendar time ${t}_{j}$^49^.

In the options_Rt.m file, we can specify the parameters related to the estimation of the effective reproduction number, namely the type of generation interval distribution (type_GId1), the mean of the generation interval (mean_GI1) and the variance of the generation interval (var_GI1) of the infectious disease of interest. For monkeypox, we employ a gamma distributed generation interval with a mean of 1.78 weeks and variance of 0.65 weeks^2^^50^ as shown below:
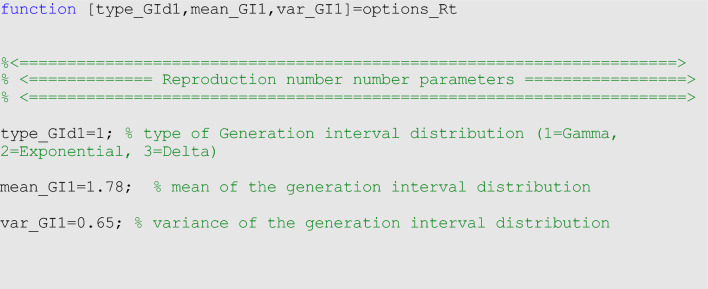


### Rolling window analysis

A rolling window analysis can be useful to assess the stability of the model parameters and forecasts over time and requires the specification of three parameters in the options_fit.m or options_forecast.m files: the start time (<tstart1>) of the first rolling window, the window size (<windowsize1>), and the end time (<tend1>) which corresponds to the start time of the last rolling window. Hence, the first rolling window contains observations for period <tstart1> to <tstart1> +  <windowsize1>− 1 , the second rolling window contains observations for period <tstart1>  + 1 through <windowsize1>, and so on. Therefore, <windowsize1> corresponds to the length of the calibration period for each model fit. The outputs obtained from the rolling window analysis correspond to the parameter estimates and their uncertainty for each rolling window subsample. A plot of the parameter estimates over the rolling windows can help examine how the estimates change with time. The parameters can be specified in the options_fit.m and options_forecast.m files as shown below:
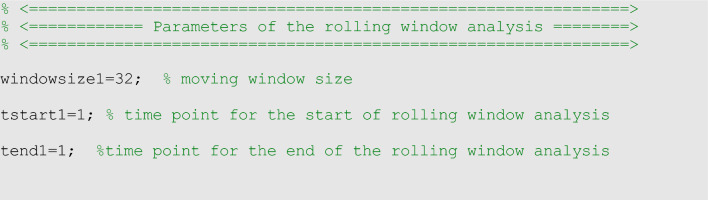


However, they can also be passed as input parameters to the fitting and forecasting functions described in the following section.

## Results and discussion

### The input dataset

For this toolbox, the time series data will be stored in the ‘input’ folder and needs to be a text file with the extension *.txt. The first column should correspond to the time index: 0,1,2,3, …, and the second column corresponds to the temporal incidence data. If the time series file contains cumulative incidence count data, the name of the time series data file must start with “cumulative”.

**Figure 4:**
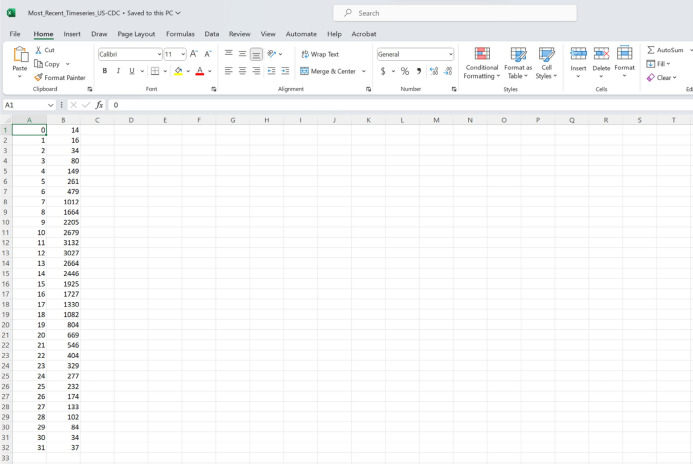
A screenshot of the weekly incidence curve of monkeypox (mpox) cases reported in the USA published by the Centers for Disease Control and Prevention (CDC) for the week of 12 May 2022 through the week of 15 December 2022^51^. The file is in the input folder within the working directory (data file path:./input/ Most_Recent_Timeseries_US-CDC.txt). The first column of the file corresponds to the time index, and the second column corresponds to the weekly incidence curve of monkeypox cases.

To illustrate the methodology presented in this tutorial paper, we employ the weekly incidence curve of monkeypox cases reported in the USA from the publicly available data published by the Centers for Disease Control and Prevention (CDC) from the week of 12 May 2022 through the week of 15 December 2022^51^. The data file is pre-loaded in the input folder within the toolbox’s working directory (data file path: ./input/ Most_Recent_Timeseries_US-CDC.txt). A snapshot in Excel of the contents of the file is shown in Fig. 4.

In the options_fit.m and options_forecast.m files, the user specifies the parameters related to model fitting and forecasting, respectively, as shown below. For instance, the parameter < cadfilename1> is a string used to indicate the name of the data file, parameter <caddisease> is a string used to indicate the name of the disease related to the time series data, and  < datatype> is a string parameter indicating the nature of the data (e.g., cases, deaths, hospitalizations).
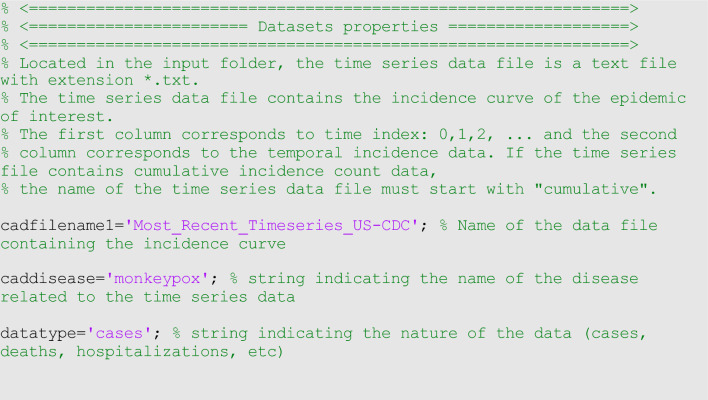


### Fitting the models to data with quantified uncertainty

The function Run_Fit_GrowthModels.m can be used to fit one of the phenomenological growth models to data with quantified uncertainty. The function uses the input parameters specified by the user in the options_fit.m file. However, the function may receive the parameters related to the rolling window analysis (<tstart1>, <tend1>, and  <windowsize1>) as passing input parameters with the remaining inputs accessed from the options_fit.m file.

For example, we can fit the generalized logistic growth model (<flag1>=1 in options_fit.m file) to the weekly incidence curve of monkeypox in the USA pre-loaded in the input folder (data file path: ./input/ Most_Recent_Timeseries_US-CDC.txt). We assume a normal error structure (i.e., <dist1>=0 in the options_fit.m file) and examine the fit of the model to the data. Since the monkeypox epidemic curve comprises 32 weeks of data, we can pass the rolling window parameters to the function call in MATLAB as follows:

 >> **Run_Fit_GrowthModels**(1,1,32).

**Figure 5:**
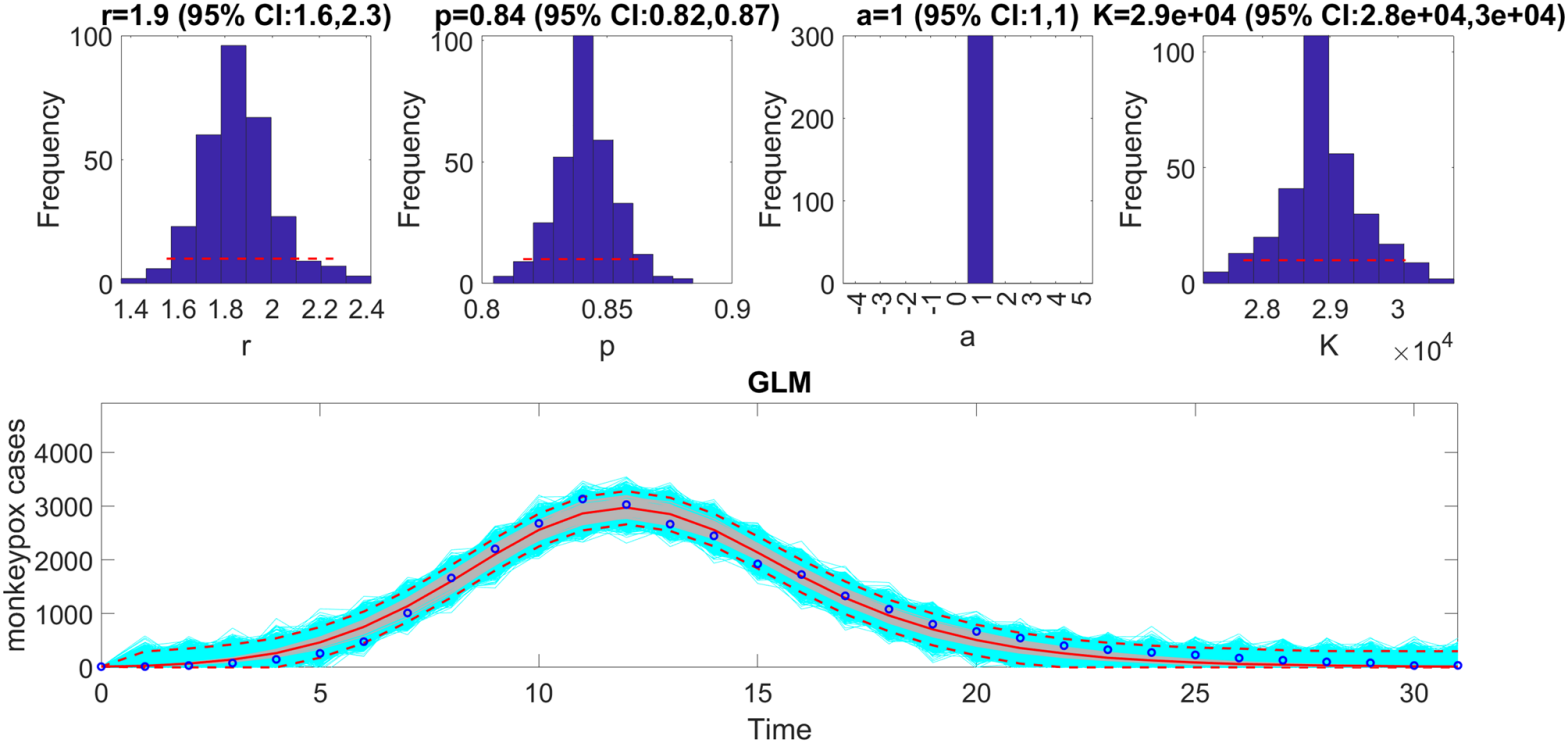
Fitting the generalized logistic-growth model (GLM) to the entire incidence curve of the monkeypox epidemic in the USA for the week of 12 May 2022 through the week of 15 December 2022. The model provides a good fit to the entire incidence curve. The model supports sub-exponential growth dynamics (i.e., *p* ~ 0.8–0.9), and has a growth rate ($$r)$$ estimated between 1.6 and 2.3. The epidemic size parameter, *K*, was estimated at ~ 28,000–30,000 cases, and the scaling parameter, $$a$$, is not estimated when employing the GLM. The horizontal dashed lines in the top panels show the range of the 95% CIs of the parameter estimates. In the bottom panel, the solid red line is the median model fit, and the dashed lines correspond to the 95% PIs. The blue dots indicate the observed data points. The gray lines correspond to the mean of the model fits obtained from the parametric bootstrapping with 300 bootstrap realizations, and the cyan lines indicate the predictive uncertainty around the model fit.

In the above call to the function, <tstart1>=1, <tend1>=1 , and <windowsize>=32. Hence, this function will generate a single model fit and store several output MATLAB files related to the model fit, parameter estimates, and the quality of model fit in the output folder. For each model fit, it will also generate a figure with the model fit and the corresponding empirical distributions of the estimated parameters (Fig. 5).

### Plot the mean model fits and compute calibration performance metrics

Once the Run_Fit_GrowthModels.m has been executed, the user can run the function plotFit_GrowthModels.m to display the model fit and the empirical distribution of the parameters. It also saves .csv files in the output folder with the model fit, the parameter estimates with 95% CIs, the Monte Carlo standard errors (MCSE) of the parameter estimates, the AIC_c_ values, the calibration performance metrics, and the estimated doubling times of the incidence trajectory. The call to the function from MATLAB's command line follows:

>> **plotFit_GrowthModels** (1,1,32)

The function uses the same rolling window parameters employed in the Run_Fit_GrowthModels.m function call. This function will store the following .csv files in the output folder:

1. The model fit to the data:
  Fit-flag1-1-i-1-fixI0-1-method-0-dist-0-calibrationperiod-32-horizon-0-monkeypox-cases.csv
2. Model parameter estimates:
  parameters-rollingwindow-flag1-1-fixI0-1-method-0-dist-0-tstart-1-tend-1-calibrationperiod-32-horizon-0-monkeypox-cases.csv
3. Monte Carlo standard errors:
  MCSES-rollingwindow-flag1-1-fixI0-1-method-0-dist-0-tstart-1-tend-1-calibrationperiod-32-horizon-0-monkeypox-cases.csv
4. AIC_c_ values:
  AICcs-rollingwindow-flag1-1-fixI0-1-method-0-dist-0-tstart-1-tend-1-calibrationperiod-32-horizon-0-monkeypox-cases.csv
5. Calibration performance metrics:
  parameters-rollingwindow-flag1-1-fixI0-1-method-0-dist-0-tstart-1-tend-1-calibrationperiod-32-horizon-0-monkeypox-cases.csv
6. Doubling times:
  doublingtimes-flag1-1-tstart-1-fixI0-1-method-0-dist-0-calibrationperiod-32-horizon-0-monkeypox-cases.csv

For this example, the resulting calibration performance metrics indicate that the 95% prediction intervals include all the data points comprising the epidemic curve of monkeypox (mpox) in the USA (i.e., coverage of the 95% PI is 100%) (Table 2). Moreover, the sequence of doubling times increased from 1.04 (95% CI: 0.94, 1.16) for the 1st doubling, 1.12 (95% CI: 1.05, 1.26) for the 2nd doubling to 3.68 (95% CI: 2.59, 7.12) for the 8th doubling time. The output also shows the probability of observing the *i*th doubling time, which decreases from about 1.0 during the first eight doublings to 0.023 for the 9th doubling. Hence, it is improbable to observe more than 8 doublings.

**Table 2:** Calibration performance metrics for a 32-week calibration period quantifying how well the fits of the generalized logistic growth model and the Richards model captured the epidemic curve of monkeypox in the USA.

Model	MAE	MSE	Coverage 95% PI	WIS
Generalized logistic growth model	110.34	17,277.54	100.00	63.39
Richards model	69.59	7595.55	100.00	43.17

The metrics indicate that the Richards model yields a better fit to the data in terms of the MAE, MSE, and WIS while both models achieved a 100% coverage of the 95% prediction interval.

**Figure 6:**
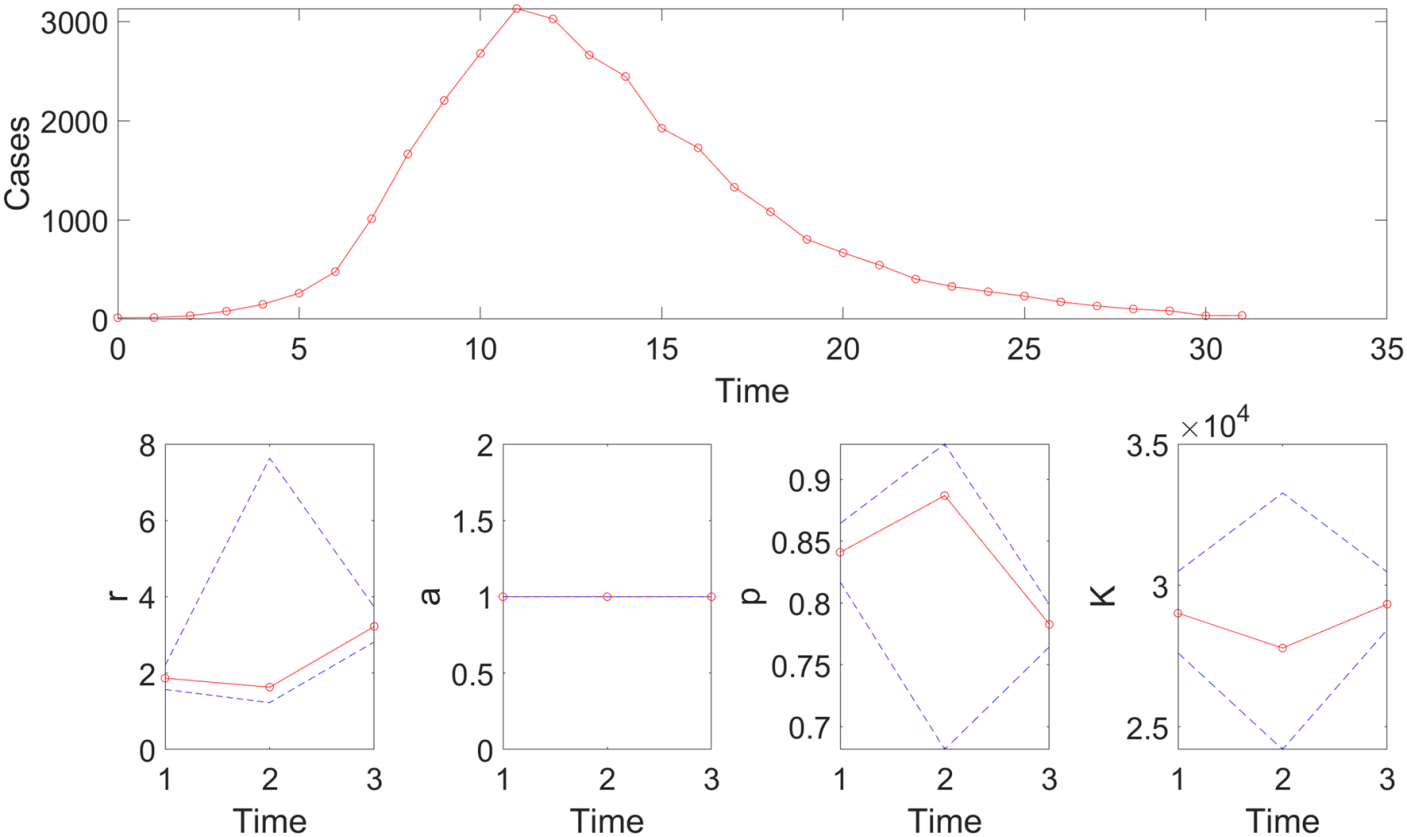
Results of the rolling window analysis after running the function plotFit_GrowthModels(1,3,30). The top panel shows the monkeypox epidemic curve in the USA for reference. The bottom panels show the temporal sequence of parameter estimates (-o-, red line), and their 95% CIs (blue dashed lines) for three different moving time windows (1–30, 2–31, and 3–32).

This function also plots the temporal sequence of parameter estimates and their uncertainty obtained from the rolling-window analysis whenever the value of the parameter <tend1> is greater than parameter  <tstart1>. For instance, after running the function Run_Fit_GrowthModels(1,3,30) to generate a rolling window analysis of model fits to capture the outbreak’s trajectory, we employed a window size of 30 with start times at 1 (end time: 30), 2 (end time: 31), and 3 (end time: 32), we can run the function plotFit_GrowthModels(1,3,30) from MATLAB’s command line to generate the rolling window analysis plot (Fig. 6).

**Figure 7:**
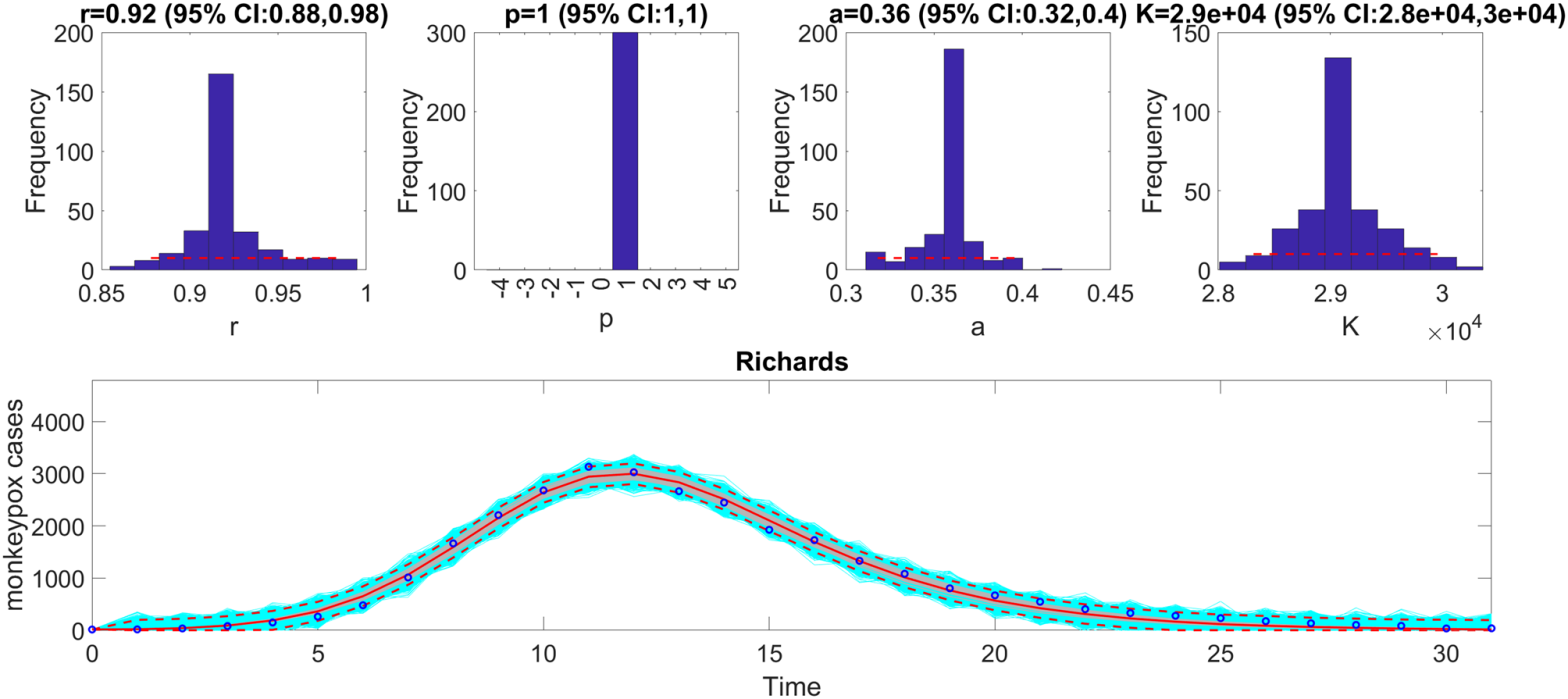
The fit of the Richards model to the entire incidence curve of the monkeypox epidemic in the USA for the week of 12 May 2022 through the week of 15 December 2022. The model provides a good fit to the entire incidence curve. The epidemic size parameter, *K*, was estimated at ~ 28,000–30,000 cases, similar to that estimated using the generalized logistic-growth model. The growth rate, $r$, was estimated to be between 0.88 and 0.98, and the scaling parameter, $a$, between 0.32 and 0.4. The growth scaling parameter, $p$, is not estimated for the Richards model. The horizontal dashed lines in the top panels show the range of the 95% CIs of the parameter estimates. In the bottom panel, the solid red line is the median model fit, and the dashed lines correspond to the 95% PIs. The blue dots indicate the observed data points. The gray lines correspond to the mean of the model fits obtained from the parametric bootstrapping with 300 bootstrap realizations, and the cyan lines indicate the predictive uncertainty around the model fit.

We can also assess the fit of the Richards model to the monkeypox incidence curve (<flag1>=4 in options_fit.m file) and compare the quality of the model fit to that obtained using the generalized logistic growth model using the performance metrics. Figure 7 shows the fit of the Richards model to the epidemic curve and the empirical distribution of the parameters.

The calibration performance metrics of the generalized logistic growth model and the Richards model (Table 2) indicate that the Richards model yields a better fit to the data in terms of the MAE, MSE, and WIS while both models achieved a 100% coverage of the 95% PI.

### Plotting and assessing model-based forecasts

To generate a forecast, we can use the function Run_Forecasting_GrowthModels.m. This function uses the input parameters provided by the user in the options_forecast.m file. However, the function can also receive  <tstart1>, <tend1>, <windowsize1>, and  <forecastingperiod>  as passing input parameters with the remaining input parameters accessed from the options_forecast.m file.

For example, we can fit the generalized logistic growth model (<flag1> =1 in options_forecast.m file) to the first 10 weeks of the monkeypox epidemic in the USA assuming a normal error structure (i.e., <dist1>  = 0 in options_forecast.m file) and generate a 4-week ahead prediction by running the function from MATLAB’s command line as follows:

 >> **Run_Forecasting_GrowthModels(**1,1,10,4**)**

**Figure 8:**
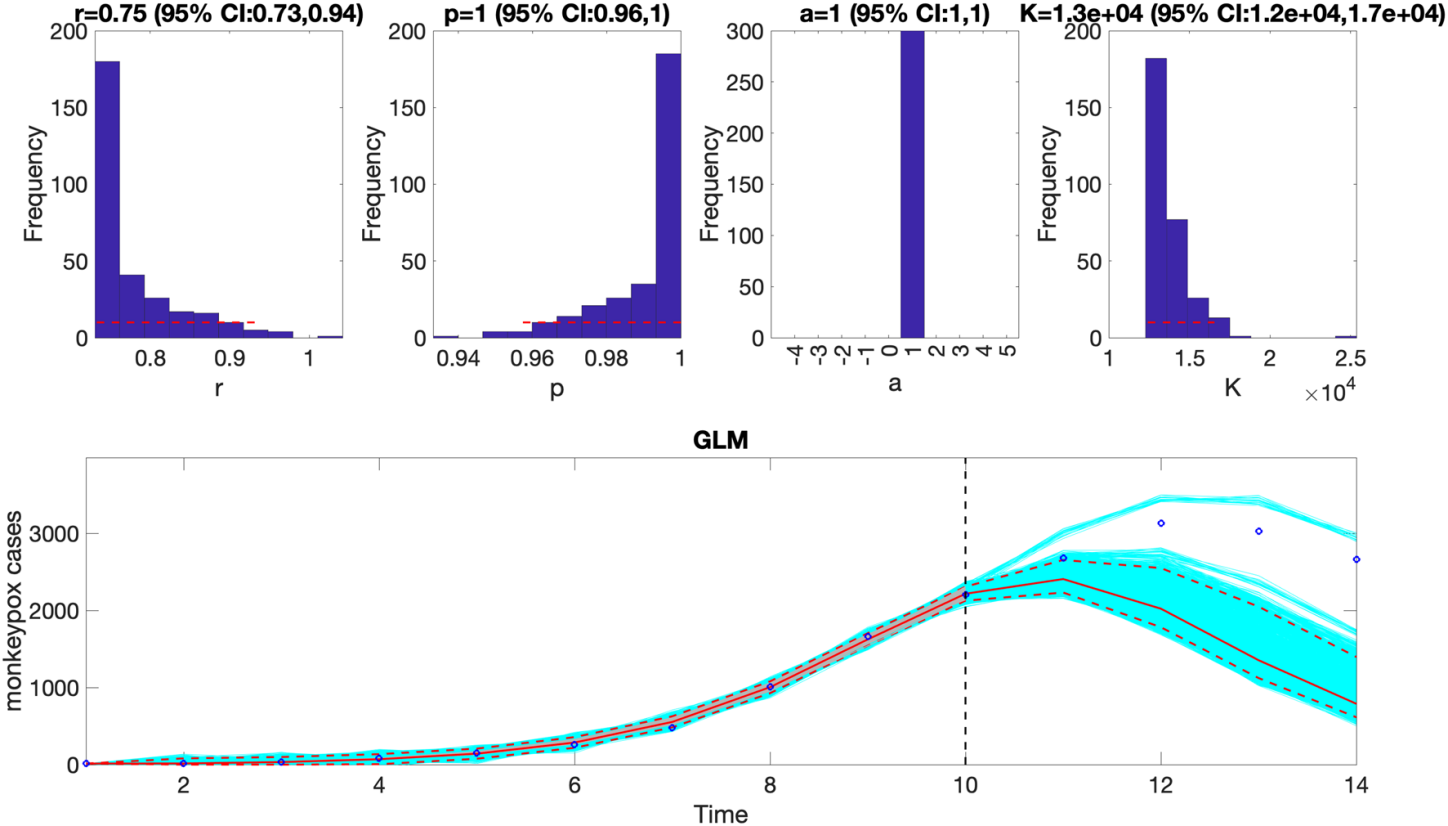
The generalized logistic-growth model (GLM) fit, and 4-week ahead forecast based on the first 10 weeks of the monkeypox epidemic in the USA for the week of 12 May 2022 through the week of 14 July 2022. The model fit is consistent with early exponential growth dynamics (i.e., *p* ~ 1), and has an estimated growth rate ($$r)$$ between 0.73 and 0.94. The epidemic size parameter, *K*, was estimated to be between 120,000 and 170,000 cases, and the scaling parameter, $$a$$, is not estimated when employing a GLM. The horizontal dashed lines in the top panels show the range of the 95% CIs of the parameter estimates. In the bottom panel, the solid red line is the median model fit, and the dashed lines correspond to the 95% PIs. The blue dots indicate the observed data points. The gray lines correspond to the mean of the model fits obtained from the parametric bootstrapping with 300 bootstrap realizations, and the cyan lines indicate the predictive uncertainty around the model fit. The vertical dashed line separates the 10-week calibration (left) and the 4-week ahead forecasting period (right). Overall, the 4-week ahead forecast underpredicted the trajectory of the epidemic.

**Figure 9:**
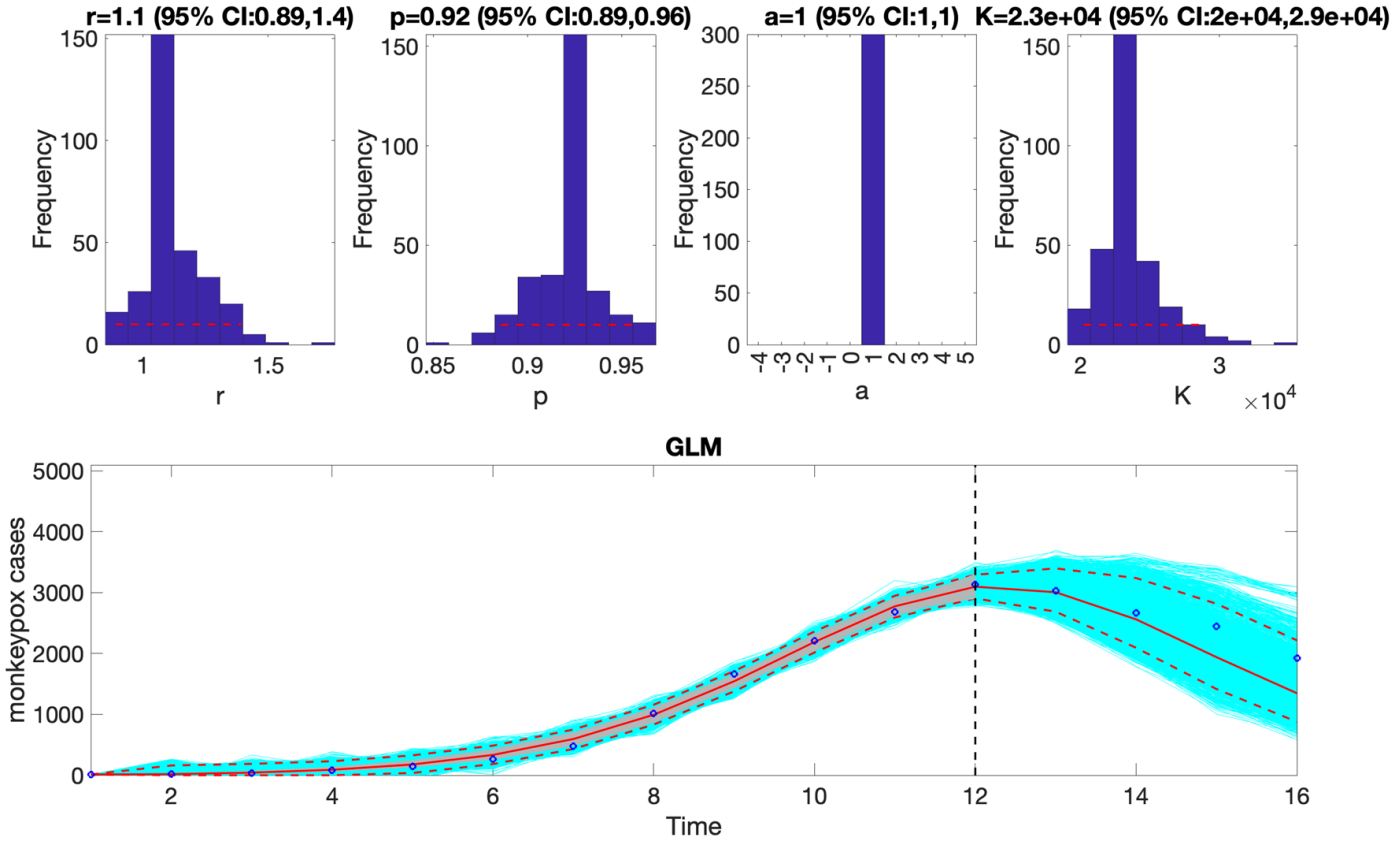
The generalized logistic-growth model (GLM) fit, and 4-week ahead forecast based on the first 12 weeks of the monkeypox epidemic in the USA for the week of 12 May 2022 through the week of 28 July 2022. The model fit is consistent with early sub-exponential growth dynamics (i.e., *p* ~ 0.92), with a growth rate ($$r)$$ estimated between 0.89 and 1.4. The epidemic size parameter, *K*, was estimated to be between 20,000 and 29,000 cases, and the scaling parameter, $$a$$, is not estimated when employing a GLM. The horizontal dashed lines in the top panels show the range of the 95% Cis of the parameter estimates. In the bottom panel, the solid red line is the median model fit, and the dashed lines correspond to the 95% prediction intervals. The blue dots indicate the observed data points. The gray lines correspond to the mean of the model fits obtained from the parametric bootstrapping with 300 bootstrap realizations, and the cyan lines indicate the predictive uncertainty around the model fit. The vertical dashed line separates the 12-week calibration (left) and the 4-week ahead forecasting period (right). Overall, the 4-week ahead forecast using the GLM with a 12-week calibration period tracked the trajectory of the epidemic well.

This function will generate a single model fit and store several output MATLAB files related to the model fit and forecast, parameter estimates, and the calibration and forecasting performance metrics. It will also generate a figure with the model fit and 4-week ahead forecast and the corresponding empirical distributions of the parameters (Fig. 8). Overall, the 4-week ahead forecast shown in Fig. 8 underpredicted the trajectory of the epidemic. Figure 1S (supplementary file 1) illustrates the results of a timing study related to the employed modeling methodology utilized in Fig. 8. For comparison, Fig. 9 also shows the model fit and 4-week ahead forecast when the model is calibrated using the first 12 weeks of the epidemic curve instead of the first 10 weeks. The 4-week ahead forecast calibrated on the first 12 weeks of data forecasted better the epidemic curve than the one calibrated on the first 10 weeks of data.

Once the user has executed the function Run_Forecasting_GrowthModels, the function plotForecast_GrowthModels can be used to plot the model-based forecast and the performance metrics of the forecast (MSE, MAE, 95% PI, WIS) based on the inputs indicated in the options_forecast.m file. However, this function can also receive  <tstart1>, <tend1>, < windowsize1>, and <forecastingperiod> as passing input parameters while the remaining inputs are retrieved from the options_forecast.m file. Moreover, the data associated with the forecasts, the parameter estimates, the Monte Carlo standard errors (MCSE) of the parameter estimates, the AIC_c_ values, the calibration and forecasting performance metrics, and the doubling times of the entire trajectory, including the forecasting period, are saved as .csv files in the output folder. For example, the following line illustrates the execution of the function from MATLAB's command window:

 >> **plotForecast_GrowthModels**(1,1,10,4)

**Figure 10:**
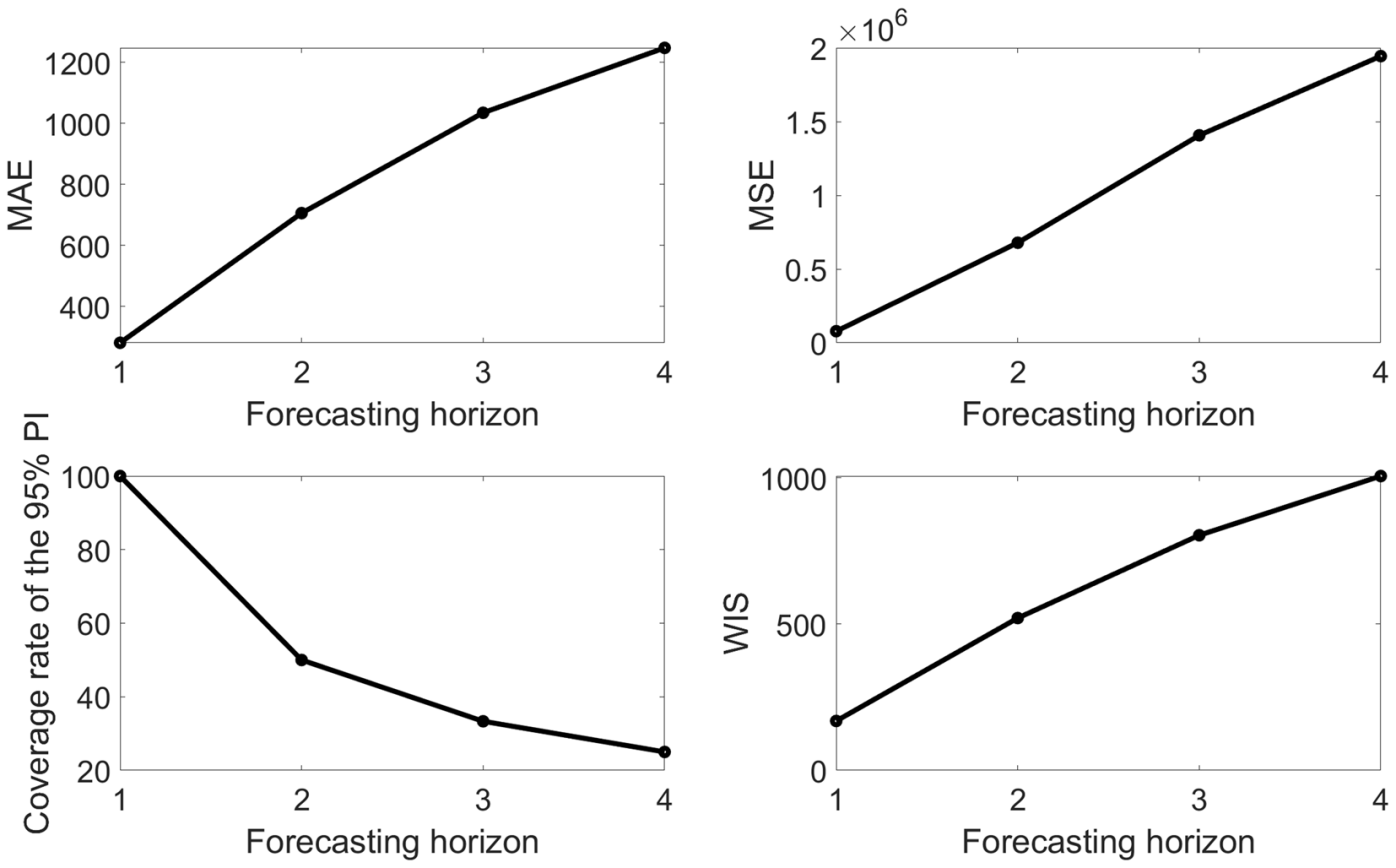
Forecasting performance metrics (MAE, MSE, 95% PI coverage, WIS) associated with the 4-week ahead forecasts obtained from fitting the generalized logistic growth model to the first 10 weeks of the monkeypox epidemic in the USA.

This function plots the model fit based on the first 10-weeks of data as the calibration period and produces a 4-week ahead forecast and the empirical distribution of the estimated parameters (Fig. 8). It also displays the associated forecasting performance metrics (Fig. 10).

**Figure 11:**
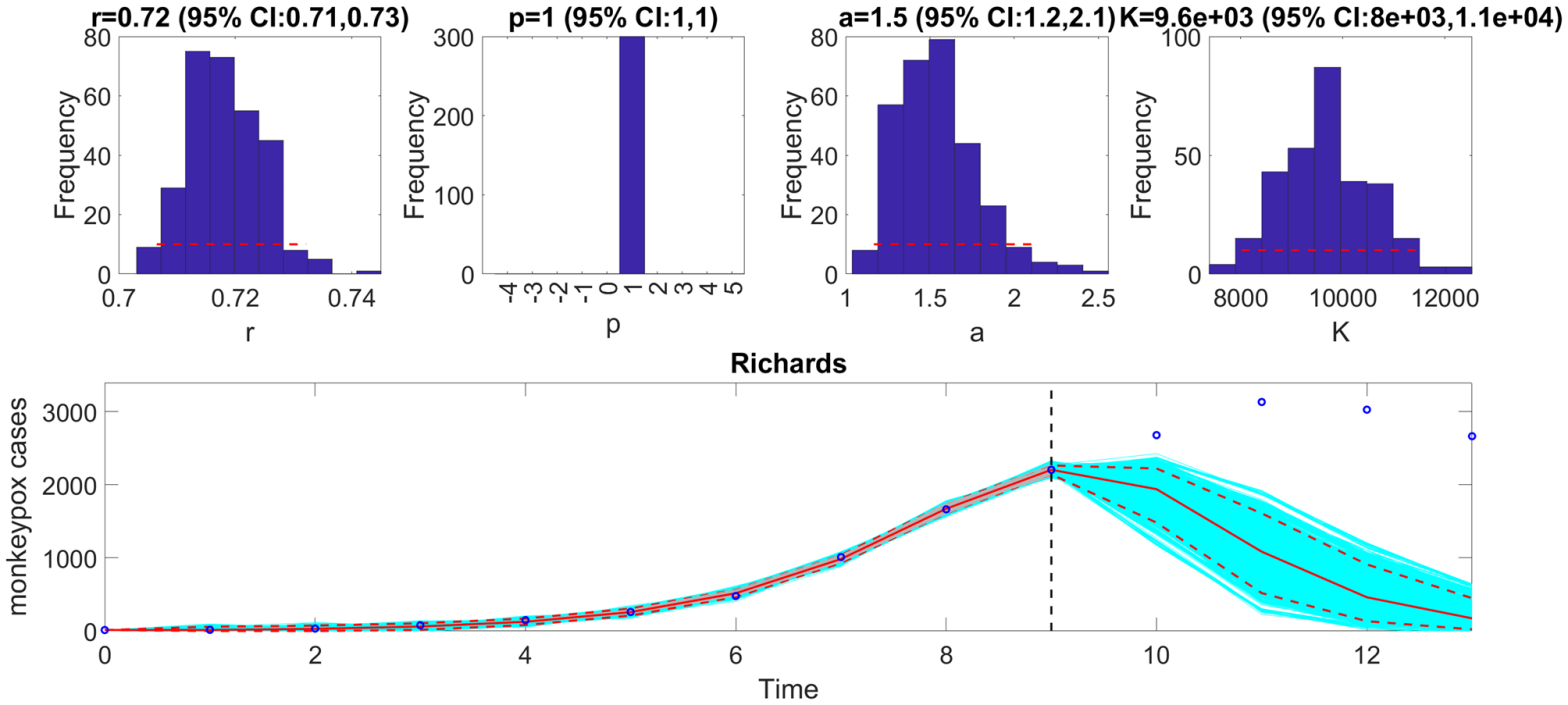
The Richards model fit, and 4-week ahead forecast based on the first 10 weeks of the monkeypox epidemic in the USA for the week of 12 May 2022 through the week of 14 July 2022. The model fit estimates the epidemic size at *K* ~ 8300–11,000 cases, and the growth rate, $r$, was estimated to be between 0.71 and 0.73. The scaling parameter, $a$, fell between 1.2 and 2.1. The growth scaling parameter, $p$, is not estimated for the Richards model. The horizontal dashed lines in the top panels show the range of the 95% CIs of the parameter estimates. In the bottom panel, the solid red line is the median model fit, and the dashed lines correspond to the 95% PIs. The blue dots indicate the observed data points. The gray lines (closely aligned with the median model fit) correspond to the mean of the model fits obtained from the parametric bootstrapping with 300 bootstrap realizations, and the cyan lines indicate the predictive uncertainty around the model fit. The vertical dashed line separates the 10-week calibration period (left) and the 4-week ahead forecast period (right). Overall, the 4-week ahead forecast underpredicted the trajectory of the epidemic.

Similarly, we can generate the forecast using the Richards model by specifying  <flag1>=4 in the options_forecast.m file and compare the forecasting performance of this model with that obtained employing the generalized logistic growth model using the performance metrics. Figure 11 shows the corresponding forecast of the Richards model based on the first 10 weeks of the epidemic curve and the resulting empirical distribution of the parameters.

The forecasting performance metrics of the generalized logistic growth model and the Richards model based on the first 10 weeks of the epidemic curve (Table 3) indicate that the generalized logistic growth model outperformed the Richards model in terms of forecasting performance.

**Table 3:** Calibration and forecasting performance metrics obtained from the fits of the generalized logistic growth model and the Richards model based on the first 10 weeks of the monkeypox epidemic in the USA forecasting out 4-weeks.

Model	MAE	MSE	Coverage 95% PI	WIS
Calibration performance				
Generalized logistic growth	18.53	813.60	90.00	13.30
Richards	11.97	300.82	100.00	8.65
Forecast performance				
Generalized logistic growth	1240.20	1,925,007.00	25.00	976.22
Richards	1960.40	4,381,564.00	0.00	1812.55

The metrics indicate that the Richards model yields a better fit to the data (better calibration performance) than the generalized logistic growth model in terms of the MAE, MSE, and WIS. However, the generalized logistic growth model outperformed the Richards model in terms of forecasting performance.

### Plotting the effective reproduction number, ${{\text{R}}}_{{\text{t}}}$

**Figure 12:**
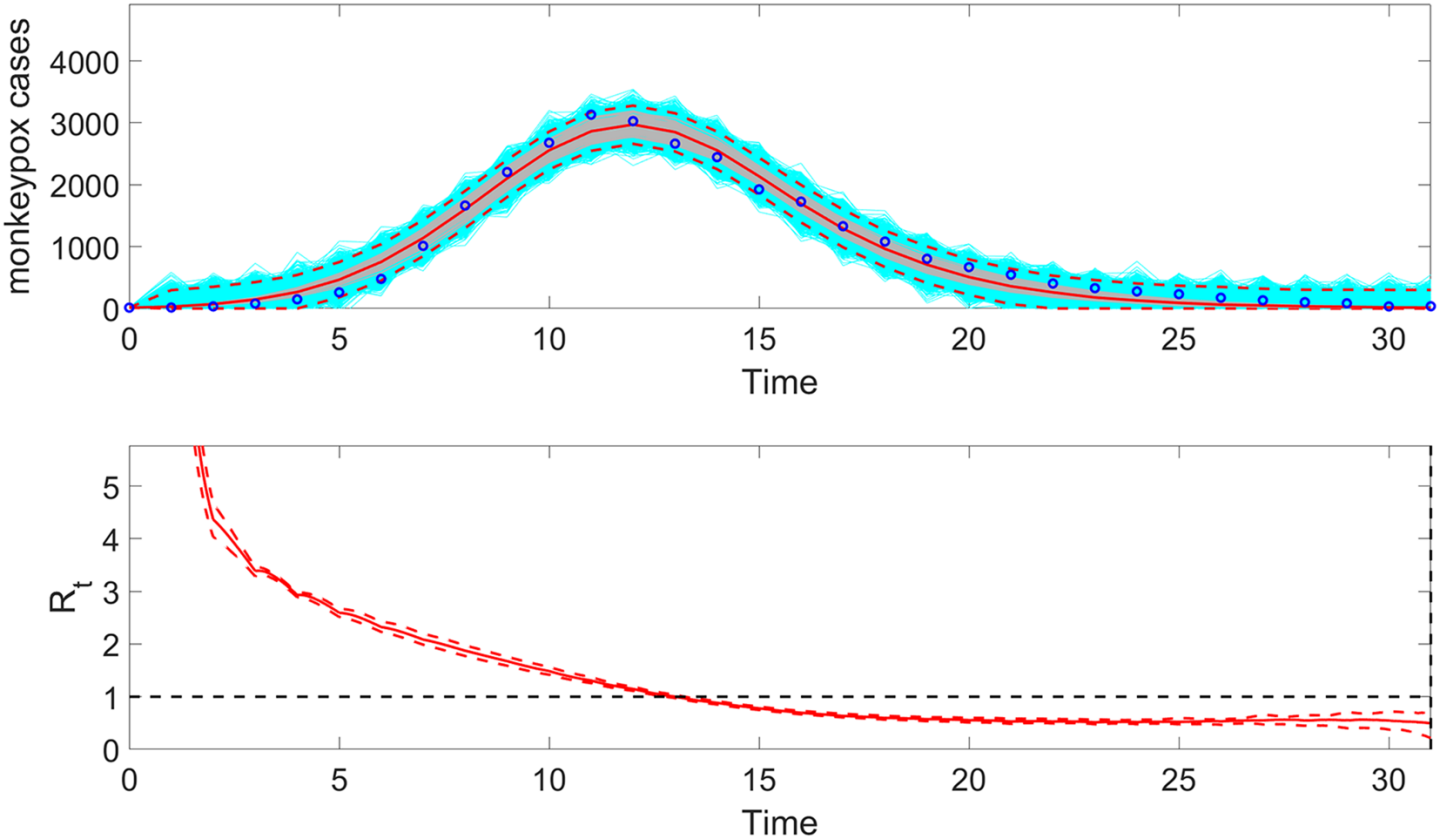
The top panel displays the fit of the generalized logistic-growth model to the entire incidence curve of the monkeypox epidemic in the USA for the week of 12 May 2022 through the week of 15 December 2022. In the top panel, the solid red line is the median model fit, and the dashed lines correspond to the 95% PIs. The blue dots indicate the observed data points. The gray lines correspond to the mean of the model fits obtained from the parametric bootstrapping with 300 bootstrap realizations, and the cyan lines indicate the predictive uncertainty around the model fit. The bottom panel shows the corresponding effective reproduction number, ${R}_{t}$**,** assuming a gamma distribution for the generation interval with a gamma distributed generation interval with a mean of 1.78 weeks and variance of 0.65 weeks^2^^50^. The solid red line corresponds to the mean effective reproduction number, while the dashed lines correspond to the 95% confidence bounds around the mean.

Once the Run_Fit_GrowthModels.m has been executed, the user can also run the function plotFit_ReproductionNumber.m to display the effective reproduction number, ${R}_{t}$ based on the inputs indicated in the options_fit.m and options_Rt.m files (Fig. 12). It also saves .csv files in the output folder with the effective reproduction number, the model fit, the parameter estimates, including 95% CIs, the Monte Carlo standard errors (MCSE) of the parameter estimates, the AIC_c_ values, the calibration performance metrics, and the estimated doubling times of the incidence trajectory. The call to the function from MATLAB's command line follows:

 >> **plotFit_ReproductionNumber(**1,1,32**)**

The function uses the same rolling window parameters employed in the Run_Fit_GrowthModels.m function call. This function will store the following .csv files in the output folder:

1. The model fit to the data:
  Fit-flag1-1-i-1-fixI0-1-method-0-dist-0-calibrationperiod-32-horizon-0-monkeypox-cases.csv
2. Model parameter estimates:
  parameters-rollingwindow-flag1-1-fixI0-1-method-0-dist-0-tstart-1-tend-1-calibrationperiod-32-horizon-0-monkeypox-cases.csv
3. Monte Carlo standard errors:
  MCSES-rollingwindow-flag1-1-fixI0-1-method-0-dist-0-tstart-1-tend-1-calibrationperiod-32-horizon-0-monkeypox-cases.csv
4. AIC_c_ values:
  AICcs-rollingwindow-flag1-1-fixI0-1-method-0-dist-0-tstart-1-tend-1-calibrationperiod-32-horizon-0-monkeypox-cases.csv
5. Calibration performance metrics:
  parameters-rollingwindow-flag1-1-fixI0-1-method-0-dist-0-tstart-1-tend-1-calibrationperiod-32-horizon-0-monkeypox-cases.csv
6. Doubling times:
  doublingtimes-flag1-1-tstart-1-fixI0-1-method-0-dist-0-calibrationperiod-32-horizon-0-monkeypox-cases.csv
7. Effective reproduction number:
  Rt-flag1-1-tstart-1-fixI0-1-method-0-dist-0-calibrationperiod-32-horizon-0-monkeypox-cases.csv

Once the user has executed the function Run_Forecasting_GrowthModels, the function plotForecast_ReproductionNumber can be used to plot the effective reproduction number associated with the entire trajectory including the forecast based on the inputs indicated in the options_forecast.m and options_Rt.m files. Moreover, the data associated with the forecasts, the parameter estimates, the doubling times, and the effective reproduction number are saved as .csv files in the output folder. For example, the following line illustrates the execution of the function from MATLAB’s command window:

 >> **plotForecast_ReproductionNumber**(1,1,10,4)

**Figure 13:**
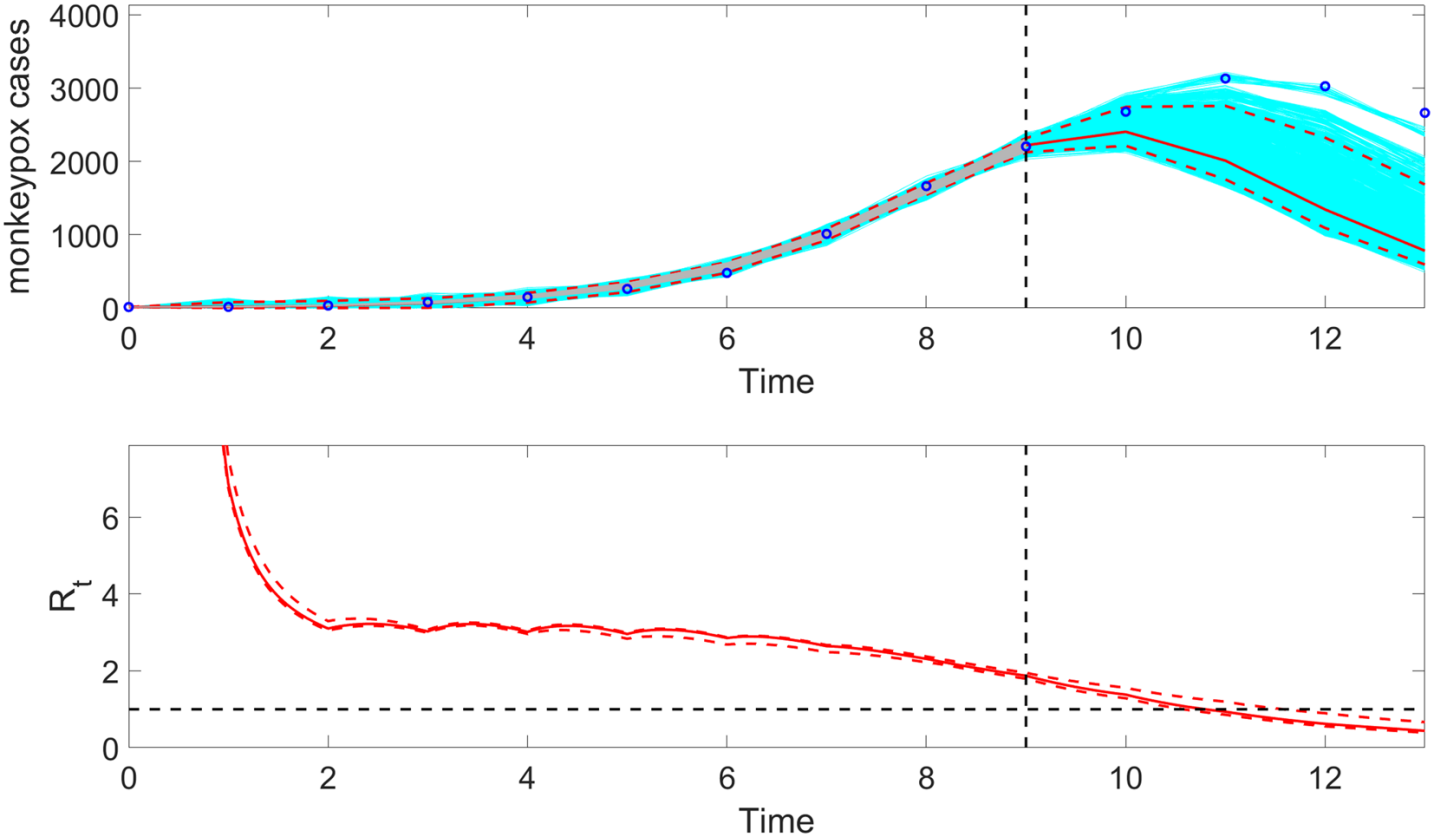
The top panel displays the generalized logistic-growth model fit, and 4-week ahead forecast based on the first 10 weeks of the monkeypox (mpox) epidemic in the USA, the week of 12 May 2022 through the week of 14 July 2022. In the top panel, the solid red line is the median model fit, and the dashed lines correspond to the 95% PIs. The blue dots indicate the observed data points. The gray lines correspond to the mean of the model fits obtained from the parametric bootstrapping with 300 bootstrap realizations, and the cyan lines indicate the predictive uncertainty around the model fit. The vertical dashed line separates the 10-week calibration period (left) and the 4-week ahead forecast period (right). The bottom panel shows the corresponding effective reproduction number, ${R}_{t}$**,** assuming a gamma distribution for the generation interval with a mean of 1.78 weeks and variance of 0.65 weeks^2^^50^. The solid red line corresponds to the mean effective reproduction number, while the dashed lines correspond to the 95% confidence bounds around the mean.

This function plots the model fit based on a 10-week calibration period, a 4-week ahead forecast, and the corresponding effective reproduction number (Fig. 13).

## Conclusion

In this tutorial-based primer, we have introduced the first toolbox, which will be broadly applicable to fit and forecast time-series trajectories using phenomenological dynamic growth models. In particular, the models included in the toolbox have been frequently applied to characterize and forecast epidemic trajectories in near real-time^10,11,14,15,18,21,28^. The toolbox can be used as part of the curriculum of student training in mathematical biology, applied statistics, infectious disease modeling, and specialty courses in epidemic modeling and time-series forecasting. It is also a helpful resource for researchers and policymakers who need to conduct short-term forecasts by relying on historical and real-time trajectory data of a process of interest.

We note some limitations and areas for future work. First, the toolbox is currently intended for users with minimal programming skills. We plan to develop a web interface with intuitive navigation to enhance the toolbox’s usability and accessibility, making it more widely accessible to users with varying technical expertise. Similarly, we plan to exploit parallel computing techniques in future software versions to speed up the running time.

### Supplementary Information

Supplementary Information.

## Data Availability

Project name: Forecasting time series with phenomenological growth models. Project home page: https://github.com/gchowell/forecasting_growthmodels. Operating system(s): Platform independent. Programming language: MATLAB. Other requirements: NA. License: This program is free software: it can be redistributed or modified under the GNU Public License as published by the Free Software Foundation, version 3 of the License. Any restrictions to use by non-academics: None.

## Abbreviations

AIC: Akaike information criterion
ARIMA: Auto regressive integrated moving average
CDC: Centers for disease control and prevention
CI: Confidence interval
GGM: Generalized growth model
GLM: Generalized logistic growth model
IS: Interval score
MAE: Mean absolute error
MLE: Maximum likelihood
MSE: Mean squared error
ODE: Ordinary differential equations
PI: Prediction interval
RIC: Richards model
GOM: Gompertz model
SARS: Severe acute respiratory syndrome
USA: United States of America
WIS: Weighted interval score
