# Supplementary material for: GrowthPredict: A toolbox and tutorial-based primer for fitting and forecasting growth trajectories using phenomenological growth models
Source: Sci Rep. 2024 Jan 18;14:1630. doi: 10.1038/s41598-024-51852-8 (PMC10796326; doi:10.1038/s41598-024-51852-8)
Supplement: Supplementary file 1 — Supplementary Information. [file 41598_2024_51852_MOESM1_ESM.pdf]

A toolbox and tutorial-based primer for fitting and forecasting growth trajectories using phenomenological growth models.

Gerardo Chowell[1*], Amanda Bleichrodt[1], Sushma Dahal[1], Amna Tariq[2], Kimberlyn Roosa[3], James M. Hyman[4], Ruiyan Luo[1]

Supplementary file 1.

Table 1S. Description of the internal functions associated with the toolbox.

Function	Role
`fit_model.m`	Fits a model to the time-series data.
`getAICc.m`	Computes the AIC_c for the model's fit.
`plotModifiedLogisticGrowthMethods1.m`	Objective function definition for fitting a model to data.
`initialParams.m`	Sets the initial guesses of the parameter values prior to model fitting.
`modifiedLogisticGrowth.m`	Defines the models for numerical solution.
`AddErrorStructure.m`	Generates realizations of the model fit with the specified error structure via parametric bootstrapping.
`computeforecastperformance.m`	Computes performance metrics for the calibration and forecasting

	periods (MAE, MSE, coverage of the 95%PI).
`computeWIS.m`	Computes weighted interval score (WIS) to assess model performance during the calibration and forecasting periods.
`getMeanVarianceRatio.m`	Computes the average of the variance-to-mean ratios from the time series data to empirically characterize overdispersion using a negative binomial distribution when `<method1>=0` and `<dist1>=2`.
`computeQuantiles.m`	Computes the quantiles from the uncertainty of the model separately for the calibration and forecasting periods.
`get_RT.m`	Computes the effective reproduction number based on the user specified parameters.
`get_nparams.m`	Determines the number of parameters estimated during the model fitting process.

`getDoublingTimeCurve.m`	Computes the doubling times based on the user specified parameters.

Supplementary Text 1S. Structure of the `options.m` file for fitting phenomenological growth models to time-series data. The coding blocks define the characteristics of the dataset, adjustments to data, the parameter estimation methods, the growth model to be fit, and the characteristics of the model.

1. Datasets properties

This section specifies the characteristics of the time series data, which will be used for inference purposes. The data file is a text file with extension *.txt, located in the input folder. The first column of the *.txt file corresponds to the time index (0,1,2,..etc.) and the second column corresponds to the incidence curve of the epidemic of interest.

While there is not a specific naming scheme that must be followed for the input data, if the time series file contains cumulative incidence count data, the name of the time series data file must start with "cumulative".

The following variables are specified in this section:

<cadilename1> This is the name of the *.txt file containing the time series data.

<caddisease>. This is a string variable indicating the name of the disease related to the time series data.

<datatype>. This is a string variable indicating the nature of the data (e.g., cases, deaths, hospitalizations).

2. Parameter estimation

This section specifies the parameter estimation method and the associated assumptions relating to the error structure in the data.

The following variables are specified in this section:

<method1>. This integer variable indicates that parameter estimation method employed to estimate the parameters from data. The following estimation methods are available:

```
method1=0; Nonlinear least squares (LSQ),
method1=1; MLE Poisson=1,
method1=3; MLE (Neg Binomial)=3, with VAR=mean+alpha*mean,
method1=4; MLE (Neg Binomial)=4, with VAR=mean+alpha*mean^2,
method1=5; MLE (Neg Binomial)=5, with VAR=mean+alpha*mean^d.
```

<dist1>. This integer variable indicates the error structure assumptions. The following error structure assumptions are available:

```
dist1=0; Normal distribution to model error structure (method1=0)
dist1=1; Poisson error structure (method1=0 OR method1=1)
dist1=2; Neg. binomial error structure where var = factor1*mean where
           factor1 is empirically estimated from the time series data (method1=0)
dist1=3; MLE (Neg Binomial) with VAR=mean+alpha*mean  (method1=3)
dist1=4; MLE (Neg Binomial) with VAR=mean+alpha*mean^2 (method1=4)
dist1=5; MLE (Neg Binomial)with VAR=mean+alpha*mean^d (method1=5)
```

<numstartpoints>. This variable defines the number of different initial guesses for the optimization procedure using Multistart in its search for the globally optimal set of parameters.

<B>. Number of bootstrap realizations utilized to characterize parameter uncertainty.

3. Growth model

This section specifies the parameters related to the growth model to be fit to the time series data.

The following variables are specified in this section:

<flag1>. An integer variable indicating the growth model that will be fit to the time-series data.

<model_name1>. The name of the model fit to the data.

<fixI0>. A Boolean variable indicating if the initial value of the time-series will be estimated or fixed according to the first data point in the time series.

4. Parameters of the rolling window analysis

This section specifies the parameters related to the calibration period or rolling window analysis.

The following variables are specified in this section:

<windowsize1> An integer variable corresponding to the length of the calibration period for each model fit.

<tstart1> An integer variable which indicates the start time of the first rolling window analysis.

<tend1> An integer variable which indicates the start time of the last rolling window analysis.

Supplementary Text 2S. Structure of the `options_forecast.m` file for fitting the specified phenomenological growth models to time-series data and producing forecasts. The coding blocks define the characteristics of the dataset, the parameter estimation methods, the growth model to be fit, and details of the rolling window analysis.

1. Datasets properties

This section specifies the characteristics of the time series data, which will be used for inference purposes. The data file is a text file with extension *.txt, located in the input folder. The first column of the *.txt file corresponds to the time index (0,1,2,..etc.) and the second column corresponds to the incidence curve of the epidemic of interest.

While there is not a specific naming scheme that must be followed for the input data, if the time series file contains cumulative incidence count data, the name of the time series data file must start with "cumulative".

The following variables are specified in this section:

<cadilename1> This is the name of the *.txt file containing the time series data.

<caddisease>. This is a string variable indicating the name of the disease related to the time series data.

<datatype>. This is a string variable indicating the nature of the data (e.g., cases, deaths, hospitalizations).

2. Parameter estimation

This section specifies the parameter estimation method and the associated assumptions relating to the error structure in the data.

The following variables are specified in this section:

<method1>. This integer variable indicates that parameter estimation method employed to estimate the parameters from data. The following estimation methods are available:

```
method1=0; Nonlinear least squares (LSQ),
method1=1; MLE Poisson=1,
method1=3; MLE (Neg Binomial)=3, with VAR=mean+alpha*mean,
method1=4; MLE (Neg Binomial)=4, with VAR=mean+alpha*mean^2,
method1=5; MLE (Neg Binomial)=5, with VAR=mean+alpha*mean^d.
```

<dist1>. This integer variable indicates the error structure assumptions. The following error structure assumptions are available:

```
dist1=0; Normal distribution to model error structure (method1=0)
dist1=1; Poisson error structure (method1=0 OR method1=1)
dist1=2; Neg. binomial error structure where var = factor1*mean where
           factor1 is empirically estimated from the time series data (method1=0)
dist1=3; MLE (Neg Binomial) with VAR=mean+alpha*mean  (method1=3)
dist1=4; MLE (Neg Binomial) with VAR=mean+alpha*mean^2 (method1=4)
dist1=5; MLE (Neg Binomial)with VAR=mean+alpha*mean^d (method1=5)
```

<numstartpoints>. This variable defines the number of different initial guesses for the optimization procedure using Multistart in its search for the globally optimal set of parameters.

<B>. Number of bootstrap realizations utilized to characterize parameter uncertainty.

3. Growth model

This section specifies the parameters related to the growth model to be fit to the time series data.

The following variables are specified in this section:

<flag1>. An integer variable indicating the growth model that will be fit to the time-series data.

<model_name1>. The name of the model fit to the data.

<fixI0>. A Boolean variable indicating if the initial value of the time-series will be estimated or fixed according to the first data point in the time series.

4. Forecasting parameters

This section specifies the parameters related to the forecast.

The following variables are specified in this section:

<getperformance>. This Boolean variable indicates whether we want to compute the forecasting performance metrics.

<forecastingperiod>. This integer variable indicates the forecast horizon (number of time units ahead).

5. Parameters of the rolling window analysis

This section specifies the parameters related to the calibration period or rolling window analysis.

The following variables are specified in this section:

<windowsize1> An integer variable corresponding to the length of the calibration period for each model fit.

<tstart1> An integer variable which indicates the start time of the first rolling window analysis.

<tend1> An integer variable which indicates the start time of the last rolling window analysis.

Code File 1S. The `options_fit.m` file corresponding to the simulated data with a Poisson error structure, initial condition $C(0) = 1$, and duration of 120 days. The three model parameters, *r*, *p* and *K* are estimated using maximum likelihood.

```
% <============================================================================>
% < Author: Gerardo Chowell  ==================================================>
% <============================================================================>
function [cadfilename1,caddisease,datatype, dist1,
numstartpoints,B,flag1,model_name1,fixI0,windowsize1,tstart1,tend1]=options_fit

% <============================================================================>
% <=========================== Declare global variables ========================>
% <============================================================================>

global method1 % Parameter estimation method

% <============================================================================>
% <================================= Datasets properties =======================>
% <============================================================================>
% Located in the input folder, the time series data file is a text file with extension
*.txt.
% The time series data file contains the incidence curve of the epidemic of interest.
% The first column corresponds to time index: 0,1,2, ... and the second
% column corresponds to the temporal incidence data. If the time series file contains
cumulative incidence count data,
% the name of the time series data file must start with "cumulative".

cadfilename1='weekly-Mpox-cases-Canada-07-19-2023'; % String variable indicating the name of
the data file containing the time-series data.

caddisease='simulated cases'; % string variable indicating the name of the disease or
subject related to the time series data

datatype='cases'; % string variable indicating the nature of the data (cases, deaths,
hospitalizations, etc)

% <=============================================================================>
% <============================ Parameter estimation ============================>
% <=============================================================================>

method1=1; % This integer variable indicates the parameter estimation method employed to
estimate the parameters from data.
% The following estimation methods are available:

% Nonlinear least squares (LSQ)=0,
% MLE Poisson=1,
% MLE (Neg Binomial)=3, with VAR=mean+alpha*mean;
% MLE (Neg Binomial)=4, with VAR=mean+alpha*mean^2;
% MLE (Neg Binomial)=5, with VAR=mean+alpha*mean^d;

dist1=1; % This integer variable indicates the error structure assumptions. The following
error structure assumptions are available:

%dist1=0; % Normal distribution to model error structure (method1=0)
%dist1=1; % Poisson error structure (method1=0 OR method1=1)
%dist1=2; % Neg. binomial error structure where var = factor1*mean where
                  % factor1 is empirically estimated from the time series
                  % data (method1=0)
%dist1=3; % MLE (Neg Binomial) with VAR=mean+alpha*mean  (method1=3)
%dist1=4; % MLE (Neg Binomial) with VAR=mean+alpha*mean^2 (method1=4)
%dist1=5; % MLE (Neg Binomial)with VAR=mean+alpha*mean^d (method1=5)
```

```
switch method1
    case 1
        dist1=1;
    case 3
        dist1=3;
    case 4
        dist1=4;
    case 5
        dist1=5;
end

numstartpoints=10; % This variable defines the number of different initial guesses for the
optimization procedure using Multistart
% in its search for the globally optimal set of parameters.

B=300; % Number of bootstrap realizations utilized to characterize parameter uncertainty.

% <==============================================================================>
% <=============================== Growth model ===================================>
% <==============================================================================>

EXP=-1;  % -1 = EXP
GGM=0;  % 0 = GGM
GLM=1;  % 1 = GLM
GRM=2;  % 2 = GRM
LM=3;   % 3 = LM
RICH=4; % 4 = Richards
GOM=5; % 5 = Gompertz


flag1=GLM; % Integer variable indicating the growth model that will be fit to the time-
series data.

model_name1='GLM';  % A string variable indicating the name of the model.

fixI0=1; % Boolean variable indicating whether initial value in the time-series will be
estimated or fix according to the first data point in the time series.

% <=================================================================================>
% <========================= Parameters of the rolling window analysis ===============>
% <=================================================================================>

windowsize1=120; % Integer variable indicating the moving window size

tstart1=1; % Integer variable indicating the time point for the start of rolling window
analysis

tend1=1; %Integer variable indicating the time point for the end of the rolling window
analysis
```

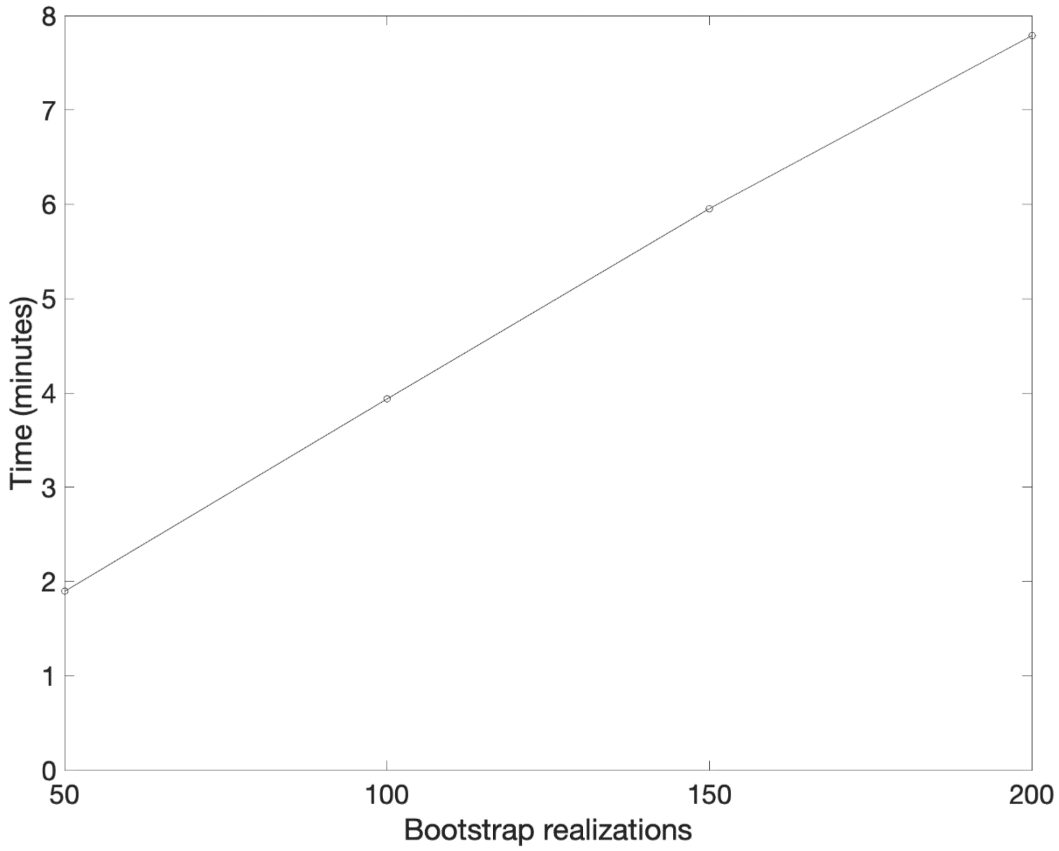

Figure 1S. The running time, as a function of the number of bootstrap realizations, to generate Figure 8 in the main manuscript. As expected, the running time to fit the model, generate the forecast with quantified uncertainty, quantify the performance metrics, and save the outputs increases approximately linearly with the number of bootstrap realizations.
